# Field validation of the performance of paper-based tests for the detection of the Zika and chikungunya viruses in serum samples

**DOI:** 10.1038/s41551-022-00850-0

**Published:** 2022-03-07

**Authors:** Margot Karlikow, Severino Jefferson Ribeiro da Silva, Yuxiu Guo, Seray Cicek, Larissa Krokovsky, Paige Homme, Yilin Xiong, Talia Xu, Maria-Angelica Calderón-Peláez, Sigrid Camacho-Ortega, Duo Ma, Jurandy Júnior Ferraz de Magalhães, Bárbara Nayane Rosário Fernandes Souza, Diego Guerra de Albuquerque Cabral, Katariina Jaenes, Polina Sutyrina, Tom Ferrante, Andrea Denisse Benitez, Victoria Nipaz, Patricio Ponce, Darius G. Rackus, James J. Collins, Marcelo Paiva, Jaime E. Castellanos, Varsovia Cevallos, Alexander A. Green, Constância Ayres, Lindomar Pena, Keith Pardee

**Affiliations:** 1 Department of Pharmaceutical Sciences, Leslie Dan Faculty of Pharmacy, University of Toronto, Toronto, Ontario Canada; 2 En Carta Diagnostics, Paris, France; 3 Department of Virology, Aggeu Magalhães Institute, Oswaldo Cruz Foundation (FIOCRUZ), Recife, Brazil; 4 LSK Technologies Inc, Kitchener, Ontario Canada; 5 Department of Entomology, Aggeu Magalhães Institute, Oswaldo Cruz Foundation (FIOCRUZ), Recife, Brazil; 6 Instituto de Virologia, Universidad El Bosque, Bogotá, Colombia; 7 Biodesign Center for Molecular Design and Biomimetics, The Biodesign Institute and the School of Molecular Sciences, Arizona State University, Tempe, AZ USA; 8 Pernambuco State Central Laboratory (LACEN/PE), Department of Virology, Recife, Brazil; 9 University of Pernambuco (UPE), Serra Talhada Campus, Serra Talhada, Brazil; 10 Wyss Institute for Biologically Inspired Engineering, Harvard University, Boston, MA USA; 11 Instituto Nacional de Investigación en Salud Pública ‘Dr. Leopoldo Izquieta Pérez’, Quito, Ecuador; 12 Department of Chemistry and Biology, Ryerson University, Toronto, Ontario Canada; 13 Institute for Biomedical Engineering Science and Technology (iBEST), a partnership between Ryerson University and St. Michael’s Hospital Toronto, Toronto, Ontario Canada; 14 Department of Biological Engineering, Massachusetts Institute of Technology, Cambridge, MA USA; 15 Institute for Medical Engineering and Science, MIT, Cambridge, MA USA; 16 Synthetic Biology Center, MIT, Cambridge, MA USA; 17 Harvard-MIT Program in Health Sciences and Technology, Cambridge, MA USA; 18 Broad Institute of MIT and Harvard, Cambridge, MA USA; 19 Department of Biomedical Engineering, Boston University, Boston, MA USA; 20 Molecular Biology, Cell Biology & Biochemistry Program, Graduate School of Arts and Sciences, Boston University, Boston, MA USA; 21 Biological Design Center, Boston University, Boston, MA USA; 22 Department of Mechanical and Industrial Engineering, University of Toronto, Toronto, Ontario Canada

**Keywords:** Synthetic biology, Riboswitches, Diagnosis, Viral infection

## Abstract

In low-resource settings, resilience to infectious disease outbreaks can be hindered by limited access to diagnostic tests. Here we report the results of double-blinded studies of the performance of paper-based diagnostic tests for the Zika and chikungunya viruses in a field setting in Latin America. The tests involved a cell-free expression system relying on isothermal amplification and toehold-switch reactions, a purpose-built portable reader and onboard software for computer vision-enabled image analysis. In patients suspected of infection, the accuracies and sensitivities of the tests for the Zika and chikungunya viruses were, respectively, 98.5% (95% confidence interval, 96.2–99.6%, 268 serum samples) and 98.5% (95% confidence interval, 91.7–100%, 65 serum samples) and approximately 2 aM and 5 fM (both concentrations are within clinically relevant ranges). The analytical specificities and sensitivities of the tests for cultured samples of the viruses were equivalent to those of the real-time quantitative PCR. Cell-free synthetic biology tools and companion hardware can provide de-centralized, high-capacity and low-cost diagnostics for use in low-resource settings.

## Abstract (web summary)

Double-blinded studies show that paper-based diagnostic tests and a companion portable device designed for use in low-resource settings perform well for the diagnosis of the Zika and chikungunya viruses in serum samples.

## Main

The 2015–2016 outbreak of the Zika virus in Latin America transformed the virus into a global concern that infected an estimated 210,000 patients and caused congenital anomalies in thousands of newborns in Brazil alone^1–6^. This public health crisis highlighted the need for rapid and low-cost testing that can be deployed beyond the reach of centralized clinical diagnostic labs. Such centralized labs use real-time quantitative PCR (RT–qPCR) for the detection of pathogens, which, although tremendously sensitive and specific, requires specialized laboratory equipment that is cumbersome and costly. The result is a sparse network of diagnostic hubs that can be difficult to scale during an outbreak, often leading to bottlenecks in testing^7,8^. This was the case in the hardest-hit country, Brazil, where RT–qPCR-based diagnostics for the Zika virus were provided by five centralized national reference laboratories, which led to limited access and delays in results^9,10^. The circumstance was worsened by overlapping clinical symptoms of the Zika virus with other endemic arboviruses^5,11,12^, cross-reactivity in antibody tests and a lack of portable antigen tests^11,13^.

Although most patients do recover from the Zika virus, the nature of mosquito-borne transmission makes this a disease of poverty, with most infections occurring in peri-urban settings, where standing water is common and public infrastructure is often inadequate. Studies following the 2016 outbreak found that inadequate surveillance and diagnostic systems contributed to spread of the Zika virus, and that social and economic disparity in access to health services exacerbated the challenges faced by vulnerable populations^14^. Taken together, the shortfall in diagnostic capacity led to calls for molecular diagnostics that can be used at the point of care (POC) and motivated the development of several new Zika diagnostic technologies, many of which came from the field of synthetic biology^15–17^.

**Fig. 1:**
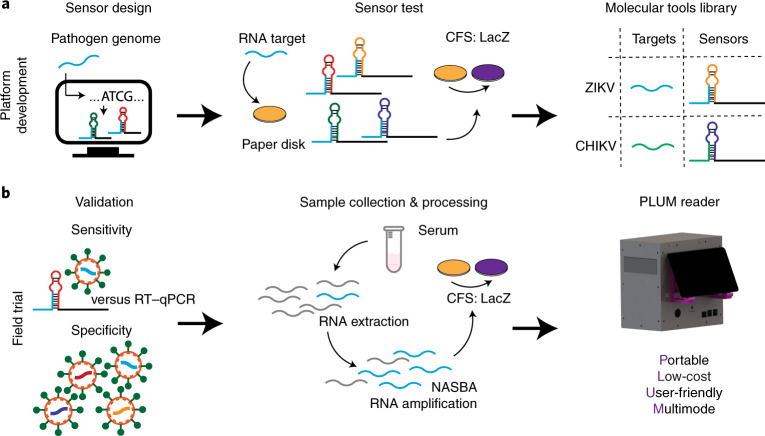
Schematic of the paper-based diagnostic system. **a**, System development. Virus-specific toehold-switch-based sensors were computationally designed based on the target virus genomic sequence. DNA encoding the toehold switch was then embedded into paper discs with a cell-free system (CFS) that transcribes the RNA-based sensor. In the presence of target viral RNA, the toehold switch enables cell-free translation of the LacZ (CFS: LacZ) reporter gene to create a colorimetric output (yellow → purple). The system is programmable and can be similarly applied to detect any pathogen sequence, enabling the formation of a molecular tools library. **b**, Field trial. Using cultured virus, the analytical sensitivity and specificity of the paper-based diagnostic were first validated using a two-step NASBA and toehold switch-based method. All of the data were collected and analysed using the in-house PLUM reader. RT–qPCR was performed in parallel for all experiments for comparison. Using the same method, validation was followed by a patient trial using RNA extracted from patient serum samples.

We were one of the groups that contributed to the effort for new and portable Zika virus diagnostics^15^. Using computationally designed toehold switch-based sensors (Supplementary Note 1) targeting the Zika RNA genome, we developed a paper-based test using cell-free protein expression reactions (PURExpress) that could be freeze-dried for distribution without refrigeration. Containing the recombinant enzymes of transcription and translation from *Escherichia coli*, these reactions first transcribe the RNA-based toehold switch from a DNA template and then, if the target Zika viral sequence is present, translate a reporter protein (for example, β-galactosidase (LacZ)) to create an optical signal through enzymatic cleavage of chlorophenol red-β-D-galactopyranoside (CPRG, yellow → purple; Fig. 1a)^18^. To reach clinically relevant sensitivity, an isothermal RNA amplification reaction (nucleic acid sequence-based amplification (NASBA)) was placed upstream in the workflow to provide detection down to the low-femtomolar range (3 fM)^19,20^. Notably, all of the molecular components for the test are independent of the PCR supply chain and, if needed, can be fabricated using the infrastructure of a simple microbiology lab^15,18,21–24^. These features make this in vitro and biosafe technology well suited for use where there is a need for de-centralized capacity, such as in low-resource settings or during public health crises.

Until recently, portable diagnostic technologies based on synthetic biology have remained largely untested under field conditions with patient samples. To address this need for validation, we assembled a team of laboratories from five countries to perform a patient trial of our paper-based Zika diagnostic devices onsite in Latin America (Fig. 1b)^15^. Here we report field trials for a synthetic biology-based diagnostic using patient samples, and we benchmarked the test performance directly to a US Centers for Disease Control (CDC) RT–qPCR test for the virus^25^. After optimization of the molecular components, and as part of establishing a PCR-free molecular diagnostic at field sites away from the benches of home laboratories in Canada and the US, we developed a low-cost, computer vision-enabled, automated plate reader (which we named the PLUM reader, for portable, low-cost, user-friendly, multimode reader) to perform low-volume, high-capacity optical measurements in a 384-well format. Using RNA extracted from the serum of patient samples, we find that the combined PLUM reader and paper-based Zika sensor provide analytical sensitivity and specificity for the Zika virus equivalent to RT–qPCR, with a diagnostic accuracy of 98.5%. We further show that the combination of molecular, hardware and software tools can be used as an adaptive system for the point of need. In a second proof-of-concept effort, we demonstrate that by simply changing the molecular components that confer specificity to the assay (in particular, NASBA primers and toehold switch), we can detect the chikungunya virus (98.5% accuracy), which is another arbovirus with worldwide distribution that causes significant morbidity^26–28^.

## Results

### Preparation of the molecular tools

**Fig. 2:**
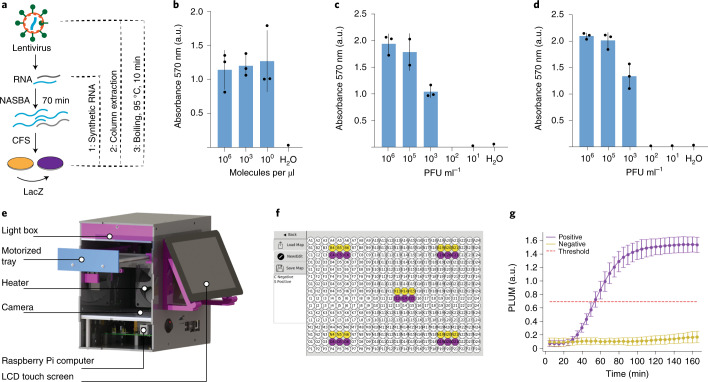
Optimization of the molecular tools for the Zika virus diagnostic test and development of the hardware and software components of the portable, low-cost PLUM reader. **a**, Schematic showing the assay workflow for tests using synthetic RNA (1), extracted RNA from lentivirus containing the target Zika virus sequence (2) and direct use of lysed lentivirus containing the target Zika RNA sequence (3). **b**–**d**, Bar graphs of analytical sensitivity determination using synthetic RNA at 1.24 × 10^x^ molecules per µl (**b**) column-extracted RNA from engineered lentivirus carrying the target RNA sequence (**c**) and heat-lysed engineered lentivirus carrying the target sequence (**d**). All data represent technical replicates from a single representative experiment (three independent biological triplicates were performed). Absorbance at 570 nm was measured on a commercial plate reader. Analysis is the mean absorbance ± s.d. of the cell-free experiments at 130 min, which were preceded by a 70 min NASBA reaction. **e**, Schematic of the PLUM reader labelled with hardware components, including Raspberry Pi computer, camera, light box, heater for incubation and an LCD touch screen for device operation. **f**, Image of the GUI showing the map setup screen for user-friendly PLUM operation and onboard data analysis. Here the map shows the plate locations of five triplicate LacZ-positive (purple) and LacZ-negative (yellow) reactions used to evaluate positional effects on data collection. **g**, Plot of PLUM reader data analysis for the spatially distributed triplicate LacZ-positive (purple) and LacZ-negative (yellow) reactions after layout in **f**. Analysis is the mean of the five selected locations; error bar represents ± s.d. of all 15 wells (Supplementary Fig. 2). The red line represents a theoretical diagnostic threshold that was determined using sensitivity data from field trials.

**Extended Data Fig. 1:**
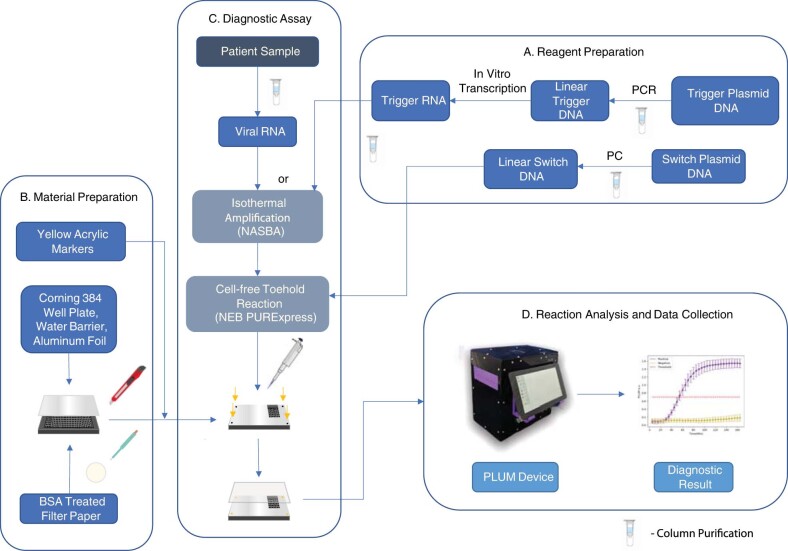
Graphical workflow. **a**, Reagent preparation: Linear DNA for toehold switches is obtained by PCR from a plasmid vector. Similarly, positive control trigger DNA is obtained from plasmids via PCR and subsequently *in* vitro transcribed into RNA. Column purification steps are indicated. **b**, Material preparation: Yellow acrylic markers are laser-cut to fit into the four corners of a 384-well plate. Paper discs are punched out from BSA-treated filter paper using a 2 mm biopsy punch and deposited in the bottom of each reaction well. The wells surrounding the reaction are then filled with water and an aluminum foil seal is placed on the plate. Using an utility knife, only the reaction wells are cut out. **c**, Diagnostic assay: Viral RNA is extracted from patient samples and then amplified through NASBA isothermal amplification, alongside positive control trigger RNA. The amplification product from NASBA is then added to cell-free reactions with linear switch DNA. Clear PCR film is applied to the plate to seal the reactions. The plate is placed inside the pre-incubated PLUM device. **d**, Reaction analysis and data collection: The PLUM software recognizes the plate and continues to capture the reaction (5 min intervals) over the next three hours. At the end of the reaction, the software analyzes the images generated, determining whether a purple to yellow color change has occurred in each well. PLUM generates a graphical report, which indicates whether the sample is positive or negative using a pre-determined threshold value.

We began by optimizing the original paper-based Zika virus diagnostic workflow for distribution to field sites (Extended Data Fig. 1)^15^. This started with evaluation of analytical sensitivity using in vitro-transcribed RNA inputs. As previous work has shown, the incorporation of an isothermal amplification step can be used to provide detection of target nucleic acids well within clinically relevant concentrations^15,17,29^. Using this two-step process, we demonstrated detection of target RNA from concentrations as low as 1.24 molecules per µl (equivalent to ~2 aM) (Fig. 2a,b and Supplementary Method 1). This is an increase in analytical sensitivity of three orders of magnitude over our previously published results, which was achieved by optimizing primer concentration (500 nM to 12.5 uM)^15^, and, ultimately, we found that reducing the primer concentration (to 500 nM) provided the best sensitivity.

Before transferring the assay to the teams in Latin America, we wanted to confirm that the workflow would accommodate the detection of virus-encapsulated target sequences. For this, we chose to test the diagnostic with an engineered lentivirus containing a small segment of the Zika viral genome. Lentivirus was chosen over the Zika virus for safety and practical reasons. Although both viruses are categorized as Biosafety Level 2 (BSL-2), the lentivirus contains only a small portion of the Zika viral genome, reducing risk of disease, and has been engineered with safety features that mitigate many of the risks of working with Zika virus, including replication incompetence^30^. These factors, combined with the lentivirus being a widely used and easy-to-implement research tool, made the virus a useful proxy to evaluate assay performance from the context of encapsulated RNA.

We began by extracting RNA from the lentiviral particles using conventional column purification (Fig. 2a,c) and then tested the ability of the diagnostic assay to detect the lentiviral RNA without the extraction step, using a simple boiling step to lyse the viral capsid for direct assay input (Fig. 2a,d). In both cases, the Zika assay was able to detect the lentivirus down to 10^3^ plaque-forming units (PFU) per ml. With the diagnostic assay ready for field testing, the primer sequences, toehold switches and protocols were transferred to laboratories in Brazil, Ecuador and Colombia to establish testing capacity onsite in Latin America.

### A portable, high-capacity plate reader

The transition of a paper-based diagnostic assay to field-deployable applications requires a field-ready companion device, and so, with no commercial options for low-cost and portable optical quantification, we developed a portable device that provides quantitative and high-throughput measurements of the paper-based Zika diagnostics onsite. Previous efforts developed companion electronic readers; however, the devices had limited sample capacity (*n* < 16) and required a laptop and separate incubator for operation^15,22^. Moreover, these designs required a dedicated light-emitting diode (LED)/sensor pair (US$6.50) for each sample, which alone would make the deployment of the high-capacity system needed for a patient trial impractical and costly for a 384-well format.

To meet this need, we designed the PLUM reader, an affordable, computer vision-enabled, camera-based plate reader, which is essentially a ‘lab in a box’ that serves as a low-cost, temperature-controlled plate reader (~US$500; Fig. 2e). In PLUM, a low-cost camera and a credit-card-sized computer are used to collect and analyse the data from the entire multiwell plate in a single image capture. This strategy substantially reduces the complexity and cost of the device and moves much of the data collection burden to the software.

#### Device configuration

The PLUM reader was assembled (Fig. 2e) using a Raspberry Pi computer, off-the-shelf electronics and rapid prototyping techniques such as three-dimensional (3D) printing and a laser-cut acrylic housing. Much like a conventional plate reader, users interact with a user-friendly graphical user interface (GUI) (Fig. 2f and Supplementary Fig. 1) that coordinates all functions of the PLUM reader. An LCD touch screen is integrated into the device for a simple and compact system that can be easily brought, as we did for this project, to multiple sites of use. This toaster-size device (20 cm × 20 cm × 20 cm) weighs only 2 kg, which compares well to conventional devices that are considerably bulkier and can weigh as much as 35 kg (for example, BioTek Neo2). The device is capable, with the correct adaptor, of operating on global power supplies or can run for 8–9 h using a standard lithium-ion brick battery (100 Wh).

PLUM software controls the 3D-printed plate reader tray, powered by a servomotor, for automated operation and dependable control over tray position (Fig. 2e). Through the GUI, the user can setup the plate map (for example, control and samples wells) for onboard data analysis and plotting at the end of each experiment (Fig. 2f,g and Supplementary Fig. 1b–d). Once the plate is loaded, the position of the plate is identified through a validation algorithm that automatically finds reusable yellow acrylic markers that sit in the four corner wells of the plate for image alignment (Supplementary Fig. 1a). Upon recognition of these markers, a digital template of the multiwell plate is used to assign the red, green and blue (RGB) channel pixel values for each well into regions of interest, which are used to create absorbance-equivalent data for all 384 wells. Collateral benefits of this approach are that PLUM’s computer vision-enabled software can be easily configured to collect optical data from any plate format, and periodic calibration of device alignment is not necessary.

#### Light path

Another challenge to operationalizing the camera-based approach was the collection of high-quality, high-sensitivity data with such a simple hardware configuration. The light box, which is located at the top of the device, sits above the multiwell plate, with the camera looking up at the colour-based paper reactions in the plate much like an illuminated-stained glass window (Fig. 2e, Supplementary Note 2, and Methods for all design and component information). Our first attempts resulted in poor data quality due to high background signal from light passing through unused empty wells in the plate (data not shown). To resolve the issues of both low dynamic range and high variability over time, we applied commercially available opaque PCR foil to block incoming light from empty wells (estimated <US$1 per sheet) (Extended Data Fig. 1). This simple and low-cost solution has the added benefit of ensuring that the unused wells remain clean for future experiments.

With the basic optical system in place, we next optimized the quality of the light source in the device. Off-the-shelf white LEDs carry warm and soft undertones with low or high blue channel biases and do not provide the broad spectrum of wavelengths needed for a colour-based analysis method^31^. Accordingly, we sourced specialized broad-spectrum white LEDs, which are used for niche applications to simulate daylight, to provide a near-ideal coverage of the visible spectrum. The 54 LEDs (US$0.95 each) were arrayed using a custom-printed circuit board to create a simple and low-cost illumination source for the PLUM reader (Supplementary Note 2). To achieve an even illumination over the plate, a custom-built, 3D-printed light box housing the LED board was assembled by using a layer of tracing paper as a diffuser. To confirm that the design was free of positional measurement effects, triplicate cell-free expression reactions with and without LacZ reporter were run at five locations in a 384-well plate (Fig. 2f,g). The level of variation across the 15 parallel positive-reaction and 15 negative-reaction time-course measurements taken by the PLUM reader was modest (Fig. 2g) and compared well to measurements taken of a replicate plate using a commercial plate reader (Supplementary Fig. 2, bottom).

#### Optical measurements

Optical absorbance measurements are a routine and key functionality for plate readers and are central to measurement of the LacZ reporter output for the Zika virus diagnostic (570 nm). To measure absorbance, conventional plate readers directly monitor the intensity of the wavelength of interest and create an absorbance value by inverting the quantified decrease in transmitted light signal (Supplementary Fig. 3a)^32^. However, without the sophisticated components of conventional plate readers, such as monochromators and optical slits, we needed an alternative approach. Our solution was to use computer-vision-based software to compensate for the simplicity of the PLUM reader’s components (for example, camera-based measurement; Supplementary Fig. 3b).

Here, rather than measuring absorbance directly, we use all the light reaching the RGB channels of the camera to measure the spectral colour shift caused by optical changes in the sample (Supplementary Fig. 2, left column). Using the colour additive theory, the resulting algorithm calculates absorbance-equivalent values by taking a ratio of the increasing colour channel over the decreasing colour channel value^33,34^. During each experiment, an image of the plate is collected at each capture interval (for example, 5 minute periods), and the RGB channel values for all well regions of interest are stored in a data file (for example, CSV). To quantify the purple reporter signal of Zika-positive samples, onboard software calculates the ratio of blue channel values over green channel values, providing similar absorbance-equivalent outputs (Supplementary Fig. 2). Once done, using well-assignment data from the plate map, data can then be automatically analysed, graphed (Fig. 2g), uploaded via the Internet to the cloud storage (Amazon Web Services S3) and shared worldwide with collaborators.

### Field trial of the diagnostic test for the Zika virus

After molecular and hardware training onsite in Latin America, we evaluated the performance of the Zika virus diagnostic system using cultured viruses (analytical specificity and sensitivity) and patient samples (diagnostic performance). As the site for the trial, we selected Recife in the Pernambuco State of Brazil, which was the epicentre of the 2016 Zika virus epidemic in Latin America. As a mosquito-borne abrovirus (for example, dengue and chikungunya), viral spread in the region was exacerbated by the many peri-urban settings, which, due to standing water, can serve as breeding grounds for mosquitoes such as *Aedes aegypti*. Selection of Recife provided an opportunity to trial the diagnostic in a location of endemic disease and near, ultimately, where we envision local and distributed health centres could use the technology.

For this phase of the project, a standardized diagnostic workflow was used in all experiments where, after RNA extraction, samples were tested using Zika virus-specific NASBA and paper-based cell-free reactions (Fig. 1b and Extended Data Fig. 1). The PLUM reader was used exclusively here and provided early and unbiased quantification of the colorimetric responses in real time. In parallel, as a gold-standard comparison, all samples were tested the same day using the RT–qPCR protocol for the Zika virus diagnosis, developed by the US CDC (cycle threshold (Ct) ≤ 38: positive and Ct > 38: negative)^25^.

**Fig. 3:**
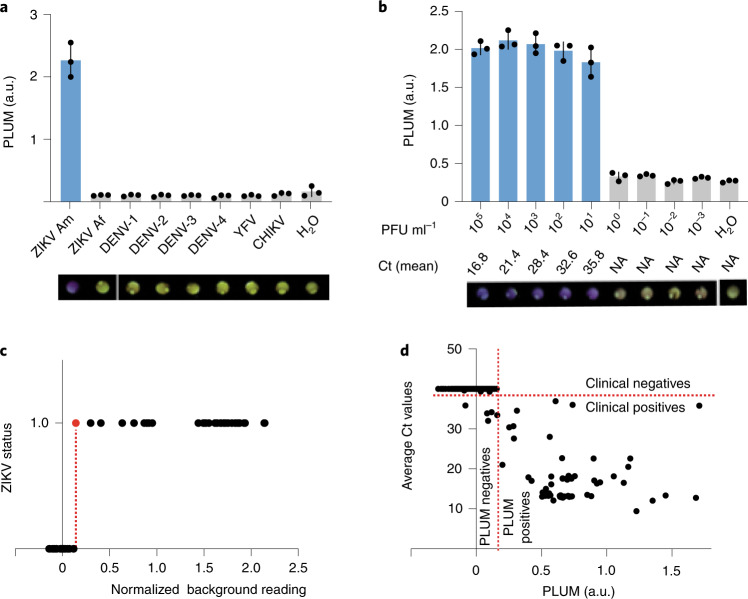
Performance of the diagnostic system for the Zika virus in Latin America. **a**, Specificity was determined for ZIKV Am against a panel of off-target viruses (at 10^6^ PFU ml^−1^) that included ZIKV Af, CHIKV, YFV and DENV-1–4. **b**, Analytical sensitivity experiment using cultured Zika virus as well as the mean Ct value obtained in the parallel RT–qPCR (Supplementary Fig. 4b). NA, not applicable. Visual outputs at final time point of 235 min are shown below the graphs in **a** and **b** (yellow, negative; purple, positive). The graphs in **a** and **b** represent the mean ± s.d. of technical replicates from a single representative experiment (three independent biological triplicates were performed) at 130 min of the cell-free experiments (preceded by 70 min NASBA reaction). RT–qPCR experiments were run in parallel for results confirmation using the CDC gold-standard assay (Supplementary Fig. 4a,b). **c**, Logistic test for threshold value determination (a.u.) of Zika virus diagnostic at 130 min, established using normalized background reading from analytical sensitivity tests performed in PLUM device. On the *y* axis, negative samples were plotted at 0; positive samples were plotted at 1. **d**, Segregation of the patient samples using the threshold value set in **c** is placed in perspective of the Ct values obtained by RT–qPCR for all 268 samples.

We began the characterization of the diagnostic system by testing the analytical specificity for the American strain of the Zika virus against a panel of seven endemic arboviruses that could, in practice, be found in patients with similar symptoms to those associated with the Zika virus. This included the chikungunya virus (CHIKV) and yellow fever virus (YFV), along with the four serotypes of the dengue virus (DENV-1–4). We also evaluated strain specificity with addition of the African strain of Zika (ZIKV Af). As can be seen by eye, a positive signal (purple colour) was only detected in the presence of the American strain of the Zika virus (Fig. 3a, bottom). The colorimetric response of reactions was quantified using the PLUM reader, with these results directly matching those from the parallel RT–qPCR assay (Fig. 3a, graph, and Supplementary Fig. 4a).

We next evaluated the analytical sensitivity of the paper-based Zika virus test. Here, we performed serial dilutions of the Zika virus after RNA extraction. The results demonstrate that the sensitivity of the paper-based diagnostic system is equivalent to the gold-standard RT–qPCR assay, with detection of the virus down to 10^1^ PFU ml^−1^ (Fig. 3b and Supplementary Fig. 4b). The difference between the maximum sensitivity observed for synthetic RNA (10^0^ RNA molecules per µl; Fig. 2b) and cultured virus (10^1^ PFU ml^−1^; Fig. 3b) can be explained by the fact that the PFU metric accounts only for infective viral particles, whereas the diagnostic sensor can detect RNA from both infective and non-infective particles. Accordingly, it is not possible to directly correlate the results from synthetic RNA (Fig. 2b) to the lentiviral data (Fig. 2c,d) or viral stock (Fig. 3b).

With the analytical specificity and sensitivity of the paper-based test established, we next set out to evaluate the diagnostic performance of the system using patient samples from suspected cases of arboviral infection collected during the 2015–2016 Zika virus outbreak in Pernambuco State, Brazil. To enable automated diagnostic analysis for users, we generated a logistic regression threshold using the time-resolved absorbance-equivalent readings from the sensitivity experiments conducted in the PLUM reader (Fig. 3c). This threshold (0.1603 arbitrary units (a.u.) above background) was then applied to the analysis of data from double-blinded, paper-based testing of extracted RNA from patient serum samples. The resulting PLUM-based analysis allowed for the differentiation of positive from negative samples as early as 130 min (Supplementary Fig. 4c). A total of 268 patient samples were analysed using our diagnostic system in parallel with RT–qPCR (Fig. 3d and Table 1). In comparison with the RT–qPCR assay^25^, we detected four false-negative and zero false-positive samples, which translates to a calculated^35^ diagnostic accuracy and specificity of 98.51% (95% confidence interval (CI), 96.22–99.59 %) and 100%, respectively. Datasets from the field trial diagnostic (0.1603 a.u) and RT–qPCR (Ct ≤ 38) were plotted to show the similar accuracy of the respective thresholds in correctly segregating positive and negative patient samples (Fig. 3d).

**Table 1:** Summary of the patient trial data

		Positive by RT–qPCR	Negative by RT–qPCR	Total	Sensitivity (%)	Specificity (%)	Accuracy (%)
ZIKV	Positive by PLUM	69	0	69	94.52 (86.56, 98.49)	100 (98.13, 100)	98.51 (96.22, 99.59)
	Negative by PLUM	4	195	199			
	Total	73	195				
CHIKV	Positive by PLUM	12	0	12	92.31 (63.97, 99.81)	100 (93.15, 100)	98.46 (91.72, 99.96)
	Negative by PLUM	1	52	53			
	Total	13	52				

For both ZIKV and CHIKV viruses, the diagnostic trial was performed using RNA extracted from patient serum. All data were collected and quantified using the PLUM reader. The patient trial data were collected over the course of 235 min. The segregation of positive and negative samples was performed at the 130 min time point for the ZIKV diagnostic using the set ZIKV threshold (Fig. 3c and Supplementary Fig. 4c) and at the 75 min time point for the CHIKV diagnostic using the set CHIKV threshold (Fig. 3d and Supplementary Fig. 6c). The 95% CIs are specified within brackets.

### Field trial of the diagnostic test for the chikungunya virus

Given the success of the Zika virus patient trial onsite in Brazil, we sought to demonstrate the versatility of the PLUM reader with programmable gene-circuit-based diagnostics. For the next diagnostic demonstration, we chose the mosquito-borne chikungunya virus, which has symptoms overlapping with the Zika virus^11,36,37^. Like the Zika virus, the chikungunya virus originated in Africa and had recently arrived in Latin America, with a sustained and ongoing spread worldwide^38^. Symptoms include fever and soreness; and, although most patients recover within weeks, severe joint pain can last for months^39^. Chikungunya infections are similarly prevalent in peri-urban and low-income communities, and so, as for the Zika virus, there is a need for accessible and de-centralized testing^40^.

**Fig. 4:**
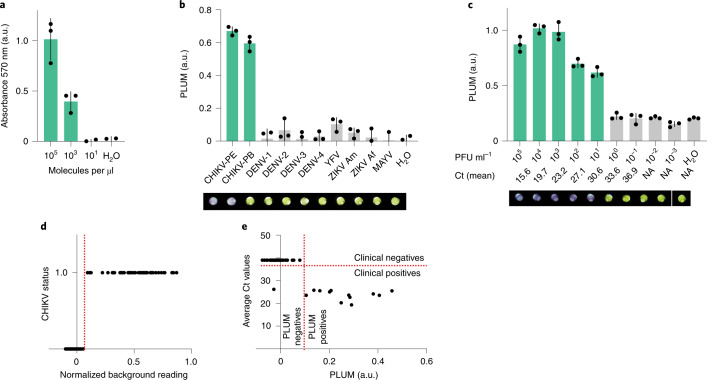
Performance of the diagnostic system for the chikungunya virus in Latin America. **a**, Analytical sensitivity using synthetic trigger RNA on the best-performing molecular sensor. Values on the *x* axis are 3.25 × 10^*x*^ molecules per µl. **b**, Analytical specificity against eight off-target arboviruses (at 10^6^ PFU ml^−1^) with the addition of MAYV. **c**, Analytical sensitivity of the test using serial dilution of extracted RNA from cultured CHIKV PE. Visual outputs (**b**, **c**) at final time point of 235 min are shown below the graphs (yellow, negative; purple, positive) as well as the mean Ct value obtained in the parallel RT–qPCR (**c** and Supplementary Fig. 6b). NA, not applicable. Analysis in **a**–**c** represents the mean absorbance ± s.d. of technical replicates from a single representative experiment (three independent biological triplicates were performed) and represent the experimental time point at 75 min of the cell-free experiments (preceded by 70 min NASBA reaction). In some cases for **a** and **b**, data points of the replicates are below zero on the *y* axis. Statistical analysis of (**b**): unpaired *t*-test, ***P = 0.0007. RT–qPCR experiments were run in parallel of (**b**) and (**c**) for results validation (Supplementary Fig. 6a,b). **d**, Logistic test for threshold value determination (a.u.) of CHIKV diagnostic at 75 min, established using normalized background reading from analytical sensitivity tests performed in PLUM device. On the *y* axis, negative samples were plotted at 0; positive samples were plotted at 1. **e**, Segregation of the patient samples using the threshold value set in **d** are placed in perspective of the Ct values obtained by RT–qPCR for all 65 samples.

As with the Zika virus diagnostic^15^, we began with the computational design of 48 toehold switches targeting various regions of the chikungunya viral RNA genome. Each toehold switch candidate was linked to the LacZ reporter and tested for detection of the corresponding synthetic target RNA sequence (2 μM; Supplementary Fig. 5). The top-performing sensor (number 10) was then optimized for analytical sensitivity using several combinations of NASBA primers specific to the region of the targeted chikungunya sequence. The combined NASBA and toehold switch-based test was able to detect the target synthetic RNA down to the clinically relevant range of 3.25 × 10^3^ molecules per µl (5 fM; Fig. 4a)^38,41^.

With the molecular components of the diagnostic validated, they were distributed to the field site in Recife, Brazil. Here, the paper-based test was evaluated for diagnostic capacity using cultured chikungunya virus (strains from Paraiba-PB and Pernambuco-PE states) and tested for analytical specificity against a panel of eight off-target endemic arboviruses (including the related Mayaro virus (MAYV)). As before, the chikungunya diagnostic test provided 100% analytical specificity for target strains (CHIKV PE, PB; Fig. 4b and Supplementary Fig. 6a). Similarly, when evaluated for analytical sensitivity using titrated chikungunya virus (strain PE), the paper-based assay provided detection down to 10^1^ PFU ml^−1^ (Fig. 4c). Although the RT–qPCR assay^38^ was more sensitive than the paper-based test (Supplementary Fig. 6b), this did not seem to limit performance with patient samples. On the basis of the analytical sensitivity data of PLUM, a chikungunya-specific threshold value (0.08 a.u. above background) was established at 75 min (Fig. 4d). Diagnostic accuracy of the chikungunya test was compared to RT–qPCR (Fig. 4e) and calculated^35^ to be 98.46% (95% CI, 91.72–99.96 %) in a double-blinded, 65-patient study, with only one false negative, and with the diagnostic sensitivity and specificity of 92.31% and 100%, respectively (Table 1, green, and Supplementary Fig. 6c).

## Discussion

This report provides the results of validation studies, onsite in Latin America, of paper-based synthetic gene networks^22,42^ as diagnostic systems for the Zika and chikungunya viruses (Figs. 3 and 4). This work also extends the concept of low-cost and portable companion hardware by describing the development of the PLUM reader. PLUM uses computer vision and an algorithm based on colour analysis, rather than optical absorbance, to provide functionality similar to conventional plate readers but at a fraction of the cost because of its simple design (Fig. 2e). The combined hardware and software of the unit provides a self-contained, automated and easy-to-use system and includes onboard data collection and analysis, incubation and cloud-based data storage.

These combined purpose-built molecular and hardware technologies come together to help move a proof-of-concept, lab-based assay towards providing low-cost, clinical-grade diagnostics at the point of need. Using 268 patient samples, we found that the paper-based Zika diagnostic could provide analytical specificity and sensitivity equivalent to RT–qPCR, with a diagnostic accuracy of 98.5% (Table 1). Similar performance for the chikungunya sensors shows promise for a path towards a generalizable diagnostic approach. The PLUM reader adds considerably to the molecular diagnostic assay by providing the option for portable use and, via signal thresholding, the automated discrimination of positive and negative samples. This translates to results as early as 2.5 h of reaction time (70 min NASBA + minimum 75 min cell-free reactions), which compares well with traditional RT–qPCR (1.5 h). Importantly, the potential for detection at the point of need could functionally reduce the time required for patient diagnosis from a scale of days to hours.

The Zika outbreak is over, yet the need for Zika virus diagnostics remains, and, according to a United Nations Development Program report, it is now expected that the Zika virus will become endemic in Latin America^14^. This prediction is supported by confirmed Zika cases in 2020, which, in Brazil alone, totalled approximately 7,000 individuals^43^. Transmission continues at low rates throughout the Americas and is currently also a concern in South Asia^44^. Although the spread of the virus is not currently an urgent concern, an estimated 2.6 billion people live in regions of the world that could establish local Zika transmission^45^, based on the presence of competent mosquito vectors and appropriate climate. By 2050, it is expected that almost half of the world’s population will live in regions of arbovirus transmission as a result of *Aedes aegypti* spread due to urbanization and climate change^46^. Thus, the ongoing need for Zika and chikungunya testing, and the risk of future surges of infection, make these viruses excellent exemplars for point-of-need diagnostics.

The PCR-free nature of our paper-based system also affords important benefits and has the potential to serve as a drop-in replacement for RT–qPCR in de-centralized applications. As we have seen with the coronavirus disease 2019 (COVID-19) crisis, the centralized nature of RT–qPCR can limit access to diagnostics and is prone to supply chain disruption during infection surges^47^. These same features, along with the challenges of cost and the logistics of shipping, also generally limit access to diagnostics in low-income and middle-income countries. Although not used here, our paper-based technology has previously been shown to be amenable to freeze-drying, for distribution without refrigeration and in-house *E. coli* cell-lysate-based reactions, for low-cost testing^15,21,22^. We see emerging diagnostics, such as the paper-based tests presented here, as having substantial near-term potential to augment existing RT–qPCR capacity, improving equity in the access to health care and aiding the responses to public health crises.

Still, important technical challenges remain to be solved ahead of practical implementation. In this report, we have provided validation results for clinical-grade molecular diagnostics using gene-circuit-based sensors and equipment that are low cost and portable. However, as with RT–qPCR, these assays still require liquid handling by skilled users to prepare samples (RNA extraction of 30 samples in about 60 min) and assays. Although we did not experience issues of cross-contamination, the need for manual intervention will increase this risk as efforts move into less technical settings. Continuation of good laboratory practices will be important as technologies make this transition.

Other real-world challenges also remain to be met. This includes ensuring that the paper-based diagnostic technology is affordable. Our estimated cost per test is US$5.48 using the price of research-grade reagents (Supplementary Table 2); however, we anticipate that this cost will be further reduced at larger scales. This compares well to the price of RT–qPCR, which, at our site in Recife (Brazil), is US$11 per reaction^48^. Notably, we also see the implementation of local and distributed reagent manufacturing (for example, NASBA and lysate-based, cell-free protein expression reactions) as crucial to realizing many of the logistic and economic advantages of distributed diagnostic tools. Work on this is already underway by others in the community, and proof-of-concept efforts show a clear path to ultra-low-cost diagnostic reagents^49–51^. Recent proof-of-concept work demonstrated onsite in Chile indicated the cost of producing the cell-free protein expression reaction (5 μl) to be US$0.069, which, here, would replace PURExpress and the related US$2.54 cost (Supplementary Table 2)^49^. Notably, access to diagnostics is not solely a consideration of cost. Reducing the burden of diagnostic testing by creating technologies that can be made using local inputs and expertise, and that are not capital intensive, will go a long way to improving public health equity in future pandemics.

The development of low-burden techniques that can de-skill sample preparation and liquid handling steps for diagnostics is also key for the practical deployment of distributed diagnostics. With this in mind, here we have demonstrated two lab-based sample preparation methods. The first, sample boiling, introduces that possibility of essentially ‘no-cost’ viral lysis. Boiling has been established by others as an effective lysis method for diagnostics^52^, and, as we found for the engineered lentivirus (Fig. 2d), boiling was also an effective lysis method for the detection of cultured Zika virus (data not shown). The second method, column-based nucleic acid purification, was used for the extraction of patient RNA from samples. At our research site in Recife (Brazil), the cost per column is US$4.96, and so this should be considered in addition to the reagent cost described above (US$5.48; Supplementary Table 2). However, most current diagnostics on the market do not include the extraction step in their kit nor in their price.

Another emerging feature for point-of-need diagnostics is the incorporation of a control sensor specific to patient RNA (such as RNase P). This control serves to guard against false-negative diagnostic results by ensuring that diagnostic samples contain RNA of sufficient quality and quantity. Although we followed gold-standard RT–qPCR methods^25,38^ at the time of our patient trial in 2019, a sample RNA control was not part of the standard practice and so was not included. False-negative results were not a significant factor in this work (Table 1); however, going forward, we envision that the implementation of such tools at the point of need will improve the utility and robustness of distributed diagnostics.

As synthetic biology moves increasingly towards the practical application of biotechnologies, this report also holds potential lessons for those seeking to validate other diagnostic systems^53^. As an early demonstration of gene-circuit-based diagnostics in the field, this work serves to inform the emerging low-cost diagnostics research community of the challenges of taking technologies from the bench and into practice in the field. This transition and the transfer of technology, as we and others have found, hold many challenges^54,55^. One main challenge was how to bring high-capacity optical characterization into new environments for distributed testing. We solved this with the development of the PLUM reader, and we anticipate that this hardware solution will help future efforts to validate and implement technologies in low-resource settings. There were also notable logistical challenges in bringing the technology from our laboratory to field sites. Accordingly, we have included brief notes that outline details that we found helpful (Supplementary Method 2). Studies that focus on the validation of technologies at the point of need will be increasingly important as the challenges of moving a technology beyond the proof-of-concept stage and towards a practical tool that can impact the world are tackled. Given the low projected cost and low technical burden for operation, we envision these and other similar technologies as a new generation of tools that will improve global access to needed diagnostics^17,56^.

## Methods

### Toehold switch and NASBA primer design method

Toehold switches were designed along with companion NASBA primers as described previously^15^. In brief, the target regions within the viral genome were analysed for suitable NASBA amplification sites based on primer sequence and thermodynamic characteristics and the secondary structure of resulting NASBA RNA amplicon. Toehold switches targeting the most promising RNA amplicons were designed and assessed based on multiple parameters, including the secondary structure of the toehold switch and the availability of the binding site in the amplified viral RNA. Combinations of toehold switches and NASBA primers predicted to provide the best performance were then selected for experimental screening.

### Toehold switch construction

Toehold switches were designed and constructed using conventional molecular biology methods. Synthetic DNA templates (Integrated DNA Technologies (IDT)) were amplified by PCR using primers (Supplementary Table 1) and cloned into a pCOLA-Duet backbone in frame with the LacZ coding sequence using Gibson assembly, as described previously^15^.

### Synthetic RNA target

DNA encoding the Zika virus target was obtained from the Collins lab in a pET15 backbone^15^. After PCR-based linearization of the template (New England Biolabs (NEB) Phusion, M0530L or NEB Q5 M0491L) using primers listed in Supplementary Table 1, in vitro T7 RNAP-based transcription of synthetic RNA was performed (NEB, E2040S). RNA samples treated with DNAse I (Thermo Fisher Scientific, K2981) were then purified (RNeasy Mini Kit, Qiagen, 74104) and used to perform toehold switch screening (2 μM RNA) or sensitivity assays (10^6^–10^0^ molecules per µl). DNA encoding the target chikungunya sequence was ordered through IDT as dsDNA with a T7 RNAP promoter. The primers used to amplify the target DNA are listed in Supplementary Table 1. After amplification, in vitro T7 RNAP-based transcription was performed as described above and then purified.

### RNA extraction virus

RNA was extracted from lentivirus samples (20 μl, 10^7^ PFU ml^−1^) or patient serum samples (140 μl) following the QIAamp Viral RNA Extraction Kit protocol (Qiagen, 52906) or by heating samples for 10 min at 95 °C. Processed RNA was then used as input to NASBA isothermal amplification reactions for use in toehold switch reactions.

### NASBA reactions

Isothermal amplification of targets was performed using a commercial NASBA kit (Life Sciences Advanced Technologies NWK-1), following the manufacturer’s instructions with slight modifications. In brief, reactions were performed in a 5 μl volume format. Per reaction, 1.67 μl reaction buffer, 0.83 μl of nucleotide mix, 0.05 μl of RNase inhibitors and 500 nM of each primer were mixed along with 1 μl of sample (water containing target RNA or sample lysate). Reactions were assembled at room temperature, incubated at 65 °C for 2 min and then at 41 °C for 10 min, before adding the enzyme mix (1.25 μl). This was followed by incubation at 41 °C for 1 h. Reactions were then added to the PURExpress reaction as previously described at a 1:7 dilution for detection of amplified target(s)^15^.

### Cell-free reactions

Cell-free reactions (PURExpress, NEB, E6800L) were assembled following the manufacturer’s instructions, as described previously^15^. Reactions were assembled to a final volume of 8 μl. In brief, 40% solution A, 30% solution B, 0.5% v/v RNase inhibitors (NEB, M0314S) and 0.2 µl of 25 mg ml^−1^ of chlorophenol red-b-D-galactopyranoside (CPRG, Roche, 10884308001) were combined. Linearized DNA encoding the corresponding toehold switch was added to cell-free reactions at 33 nM. For reactions that included isothermally amplified RNA, NASBA products were added at a 1:7 dilution (1.14 μl). The time point data presented (Figs. 2b–d, 3a,b and 4a–c) reflect the respective optimal time required for each cell-free diagnostic assay (ZIKV, 130 min; CHIKV, 75 min).

### Lentivirus engineering, cloning, culture, purification and titration

#### Plasmids

(1) pUltra-hot, third-generation lentiviral vector for bi-cistronic expression of mCherry and the gene of interest; pUltra-hot was a gift from Malcolm Moore (Addgene, plasmid 24130; http://n2t.net/addgene:24130; RRID:Addgene_24130). (2) pMD2.G, a VSV-G envelope-expressing plasmid; pMD2.G was a gift from Didier Trono (Addgene, plasmid 12259; http://n2t.net/addgene:12259; RRID:Addgene_12259). (3) pMDLg/pRRE, a third-generation lentiviral-packaging plasmid, contains Gag and Pol; pMDLg/pRRE was a gift from Didier Trono (Addgene, plasmid 12251; http://n2t.net/addgene:12251; RRID:Addgene_12251). (4) pRSV-Rev, a third-generation lentiviral-packaging plasmid, contains Rev; pRSV-Rev was a gift from Didier Trono (Addgene, plasmid 12253; http://n2t.net/addgene:12253; RRID:Addgene_12253).

#### Cloning protocol

Trigger sequence corresponding to the Zika-virus-specific toehold switches was placed downstream of the mCherry sequence between the P2A and T2A sequences^57^. The final sequence was mCherry-P2A-T3T8-T2A with the plasmid named pUltra-hot-mCherry-T3T8 (at 5093–5384 bp from ORI).

#### Cell culture

HEK293T cells were grown at 37 °C at 5% CO_2_ in a humidified incubator in DMEM or DMEM supplemented with 4,500 mg l^−1^ of glucose, ʟ-glutamine and sodium bicarbonate (D5796, Sigma-Aldrich), 10% foetal bovine serum (FBS) and 1% penicillin–streptomycin, approximately, 100 U ml^−1^ penicillin and 100 µg ml^−1^ (Gibco).

#### HEK293T transfection with lentiviral constructs

Once at 70% confluency in a 10 cm plate, cells were transfected with polyethylenimine (PEI, 408727, Sigma-Aldrich) by combining a ratio of DNA:PEI at 1:3, following manufacturer’s instructions and incubated overnight at 37 °C^58^. The ratio of plasmid DNA (µg) used was pUltra-hot-T3T8: pMD2.G: pMDLg/pRRE: pRSV-Rev = 4.5: 1.5: 3: 3. Media were then replaced with 10 ml of fresh culture media and incubated at 37 °C. Once mCherry expression was detected, media were collected and centrifuged at 1,000*g* for 5 min to pellet the debris and the supernatant was syringe filtered using a 0.4 µm filter (Millipore).

#### Lentivirus concentration

LentiX (TaKaRA, catalogue no. 631231, lot no. 1705016a) concentrating solution was added to the filtered supernatant in a 1:3 ml ratio (1 ml of LentiX to 3 ml of virus supernatant), and concentration was performed following manufacturer’s instructions.

Virus titration: HEK293T cells were plated at 3 × 10^4^ cells per well. Serial dilution of lentivirus aliquot was performed in PBS, with 5–7 technical replicates for each, and 25 µl of the diluted virus was transferred onto the wells containing the cells. The plate was placed at 37 °C for overnight incubation.

#### Median tissue culture infectious dose

Plates were analysed for cytopathic effect, and conversion from median tissue culture infectious dose (TCID_50_) to PFU was done using the calculation suggested by the American Type Culture Collection (https://www.atcc.org/support/technical-support/faqs/converting-tcid-50-to-plaque-forming-units-pfu#:~:text=For%20any%20titer%20expressed%20as,mean%20number%20of%20PFU%2Fml).

### Virus strains, virus culture and purification

All arboviruses used in this study were provided by the Laboratory of Virology and Experimental Therapy, Oswaldo Cruz Foundation (FIOCRUZ), in Recife, Brazil.

Zika virus, American strain PE243 (GenBank accession no. KX197192), was isolated from an infected patient in Pernambuco State, Brazil. Zika virus, African strain MR766 (GenBank accession no. AY632535), was isolated from infected mouse brain suspension. All four Dengue virus (DENV (1–4)) serotypes used were isolated from patient samples in Pernambuco State, Brazil: DENV-1, strain PE/97-42735 (GenBank accession no. EU259529), DENV-2, strain PE/95-3808 (GenBank accession no. EU259569), DENV-3, strain PE/02-95016 (GenBank accession no. KC425219) and DENV-4, strain PE/10-0081 (unpublished). Yellow fever virus, strain 17DD (GenBank accession no. DQ100292) is used as a vaccine strain. Chikungunya virus, strain PE2016-480 (unpublished), was isolated from a patient serum in Pernambuco State, Brazil, and chikungunya strain PB302 (unpublished) was isolated from serum of patient in Paraiba State, Brazil. Mayaro virus, strain BR/Sinop/H307/2015 (GenBank accession no. MH513597.1) was provided by the Federal University of Mato Grosso in Sinop, Brazil.

### Arbovirus cultures and titration

All viruses used in this study were propagated in Vero cells using DMEM (Gibco) supplemented with 2% of inactivated FBS (Gibco), 2 mM ʟ-glutamine (Gibco), 100 units per ml of streptomycin and 100 μg ml^−1^ of penicillin (Gibco) at 37 °C under 5% CO_2_. Mosquito-borne viruses, including ZIKV American lineage, ZIKV African lineage, DENV-1–4, YFV, MAYV, CHIKV PE and CHIKV PB, were titrated using plaque assay with titre ranging from 10^6^ to 10^7^ PFU ml^−1^. Virus stocks were stored at −80 °C before downstream applications.

### Analytical sensitivity experiment

Viral RNA from stock of all viruses (at 10^6^ PFU ml^−1^) was extracted using the QIAamp Viral Mini Kit (Qiagen, 52906) and eluted in 60 µl of water. After serial dilution of the extracted RNA (from 10^5^ to 10^−3^ PFU ml^−1^), samples were assayed in parallel with the gold-standard RT–qPCR and the NASBA/cell-free reactions^25,38^. ZIKV American strain (PE243) and CHIKV strain (PE2016-480) were the strains used in those studies.

### Analytical specificity experiment

Analytical specificity of Zika virus or chikungunya virus was determined against a panel of different mosquito-borne viruses endemic in Latin America. Extracted viral RNAs of DENV-1–4, YFV, ZIKV American, ZIKV African, MAYV, CHIKV PE and CHIKV PB at 10^6^ PFU ml^−1^ were used as inputs to NASBA/cell-free reactions and parallel RT–qPCR.

### RT–qPCR for ZIKV and CHIKV

ZIKV and CHIKV RT–qPCR was performed according to protocols established by the CDC, with minor modifications^25,38^. Reactions were performed using the QuantiNova Probe RT–PCR kit (Qiagen) following the manufacturer’s protocols for 10 μl of final volume with primers of both viruses in 800 nM final concentration and probes in 100 nM final concentration. Primers and probes for ZIKV and CHIKV can be found in Table 1. Reactions were carried out in a QuantiStudio 5 system (Applied BioSystems) with a thermal cycle programme consisting of a single cycle of reverse transcription for 15 min at 45 °C, followed by 5 min at 95 °C for reverse transcriptase inactivation and DNA polymerase activation, and then 45 cycles of 5 s at 95 °C and 45 s at 60 °C. All samples were tested in duplicates, with negative controls (all reagents except RNA) and positive controls (RNA extract from viral stock). For results analysis, the QuantStudio Design and Analysis Software version 1.5 was used with automatic threshold and baseline. Samples were considered positive with Ct ≤ 38.0.

### PLUM hardware

The PLUM reader consists of two chambers housed within a laser-cut acrylic box fastened together with metal screws and mounting brackets (Fig. 2; McMaster-Carr, 8505K14 and 98164A061; Digi-Key, 36-621-ND). The light box is designed with 54 LEDs (YJ-VTC-5730-G01-65, YUJI LED) for illumination. The motorized tray is designed to hold a standard multiwell plate and positions the plate at the correct focal length for the camera (US$35, Raspberry Pi V2, 8-megapixel). This configuration allows for clear visualization of the plate from below for capture of the image data (Supplementary Fig. 3). On the side panels, the detection chamber houses a fan-based air incubator (Incubator Warehouse, IncuKit MINI) for heating, DS18B20 temperature sensor for recording (RobotShop, RB-Dfr-270), two tray rail guides, a Hitec HSR-1425CR continuous servomotor with 12T Metal Servo Gear (RobotShop, RB-Hit-78, RB-Sct-458) for controlling the tray and a microswitch (RobotShop, RB-Tam-71) for homing the tray. The servomotor gears engage with 3D-printed tray teeth to open and close the tray.

The electronic components of PLUM are controlled by a Raspberry Pi 3B. The camera (Raspberry Pi Camera Module V2) in the detection compartment and the LCD display unit (Raspberry Pi Touch Display 7 inch) mounted on the front panel of the PLUM are connected directly to the Raspberry Pi unit with flex cables (Flex Cable for Camera-24 inch/610 mm). The Raspberry Pi controls the detection unit components, with the exception of the incubator, via a 40-pin header ribbon cable connected to the custom-made motherboard PCB (printed circuit board) (Supplementary Information, vendor). The motherboard PCB facilitates connections of the servos, temperature probe, required resistors, microswitch, power to the LCD display and light box operation with mating connectors. The device contains a power jack that can be mated with a 12V, 7A power supply or a portable battery (TalentCell Rechargeable, 100 Wh) to support 8–9 h of operation in the field.

All of the information required for the assembly of the PLUM reader is supplied as Supplementary Information. Data files for laser cutting, 3D printing and circuit diagrams and the Gerber files required for printed circuit board manufacturing can be accessed at https://github.com/PardeeLab/zikaproject_hardware.

### PLUM software

A GUI operating on Rasbian was designed using Python 2.7 and open-source APIs (Supplementary Information). The Amazon Simple Storage Service provided by Amazon Web Services (https://aws.amazon.com/) was used to scale up the storage space of PLUM through a web service interface. GUI-related and workflow-related code can be found at https://github.com/PardeeLab/zikaproject_plumcode.git.

### Statistics

Statistical analyses for analytical sensitivity experiments were performed on GraphPad Prism 7 (GraphPad Software) using an unpaired *t*-test.

### Diagnostic performance

Final accuracy tests were established using an online mathematical tool provided by MedCalc (https://www.medcalc.org/calc/diagnostic_test.php)^48^.

### Establishing threshold and data analysis

All of the sample data collected in PLUM were normalized by subtracting the negative control values (extracted water) in each run. A logistic test was run on data collected for test sensitivity analysis with Zika virus patient samples. According to the time-based thresholds, each patient data point was classified as positive or negative (Supplementary Figs. 4 and 6).

### Patient sample collection

This study was approved by the FIOCRUZ-PE institutional review board (IRB) under protocol 80247417.4.0000.5190 and research ethics board (REB) approval number at the University of Toronto (protocol 39531) and was conducted in accordance with relevant regulations and guidelines, including the ethical principles for medical research involving human subjects designed by the World Medical Association Declaration of Helsinki. Patient samples were obtained from suspected arbovirus infections, from patients who presented arthralgia, fever, exanthema and other related symptoms in an endemic area of several arboviruses in Latin America. Informed consent of all individuals included in this study was waived by the FIOCRUZ-PE IRB for diagnostic specimens.

### Reporting Summary

Further information on research design is available in the Nature Research Reporting Summary linked to this article.

## Supplementary information

Supplementary Information Supplementary methods, figures, tables and references.

Reporting Summary

Supplementary Data Nucleic acid sequences.

## Data Availability

The main data supporting the results in this study are available within the paper and its Supplementary Information. All the experimental raw data are available for research purposes from the corresponding author on reasonable request.

## Code availability

Code for controlling the device and conducting experiments on the PLUM reader can be found at https://github.com/PardeeLab/zikaproject_plumcode.git. Additional code is available from the corresponding author on reasonable request.
