# Supplementary material for: Field validation of the performance of paper-based tests for the detection of the Zika and chikungunya viruses in serum samples
Source: Nat Biomed Eng. 2022 Mar 7;6(3):246–56. doi: 10.1038/s41551-022-00850-0 (PMC8940623; doi:10.1038/s41551-022-00850-0)
Supplement: Supplementary file 1 — Supplementary methods, figures, tables and references. [file 41551_2022_850_MOESM1_ESM.pdf]

nature portfolio

https://doi.org/10.1038/s41551-022-00850-0

Supplementary information

Field validation of the performance of paper-based tests for the detection of the Zika and chikungunya viruses in serum samples

In the format provided by the
authors and unedited

Contents

Figures

Supplementary Fig. 1: PLUM software application graphical user interface (GUI).

Supplementary Fig. 2: Comparison of the PLUM (left panel) versus the commercial plate reader (right panel) for quantitative readings of the assays.

Supplementary Fig. 3: Schematic comparing the optical design of PLUM versus a conventional plate reader.

Supplementary Fig. 4: RT-qPCR data for Zika virus detection and threshold design.

Supplementary Fig. 5: Data from screening of candidate chikungunya virus toehold switch-based sensors.

Supplementary Fig. 6: RT-qPCR data for CHIKV detection and threshold design.

Table 1: Sequences

Table 2: Cost analysis of the diagnostic test

Note 1: Toehold switch background information

Method 1: RNA copy number calculation

Method 2: Documentation for International Field Work

Note 2: Hardware: Supplemental Files for Electronic Optical Reader

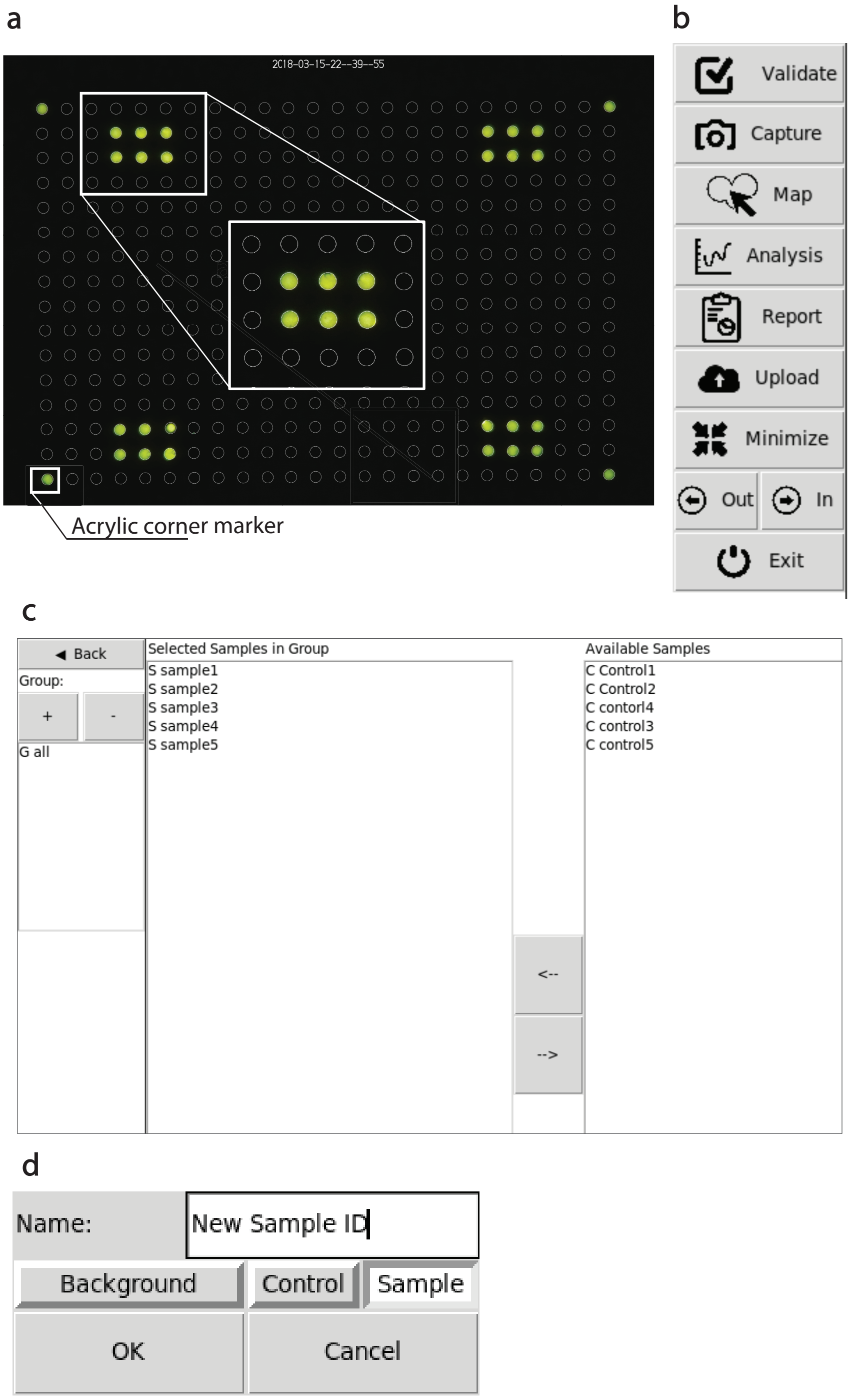

Supplementary Fig. 1. PLUM software application graphical user interface (GUI). a, Automated well alignment. Image of a 384-well plate captured from the bottom by the PLUM camera. Regions of interest (ROIs) are automatically identified by the PLUM software following plate validation. Here, grey circles indicate the ROIs that were identified following alignment of the four yellow acrylic markers in the corner wells of the plate. **b**, Image of the GUI's Launch page in the PLUM software application. The panel contains buttons to initiate tasks including plate validation, image capture, map setup, report generation, cloud upload, window minimization, move tray track and program termination. **c**, Map page of PLUM GUI for subgrouping samples. For automated data analysis and real-time plotting of data on the display, samples can be divided into subgroups (e.g., Sample, Control). New subgroups can be created or deleted using "+" and "-" buttons. Sample ID can be chosen from Available Samples pool (right) and assigned to the Selected Samples in Group pool (left) for subgrouping with arrow buttons. **d**, Map page of PLUM GUI for Sample ID assignment. Here, sample identification and type (i.e., Background, Control and Sample) can be assigned to corresponding well locations (Supplementary Fig. 1a).

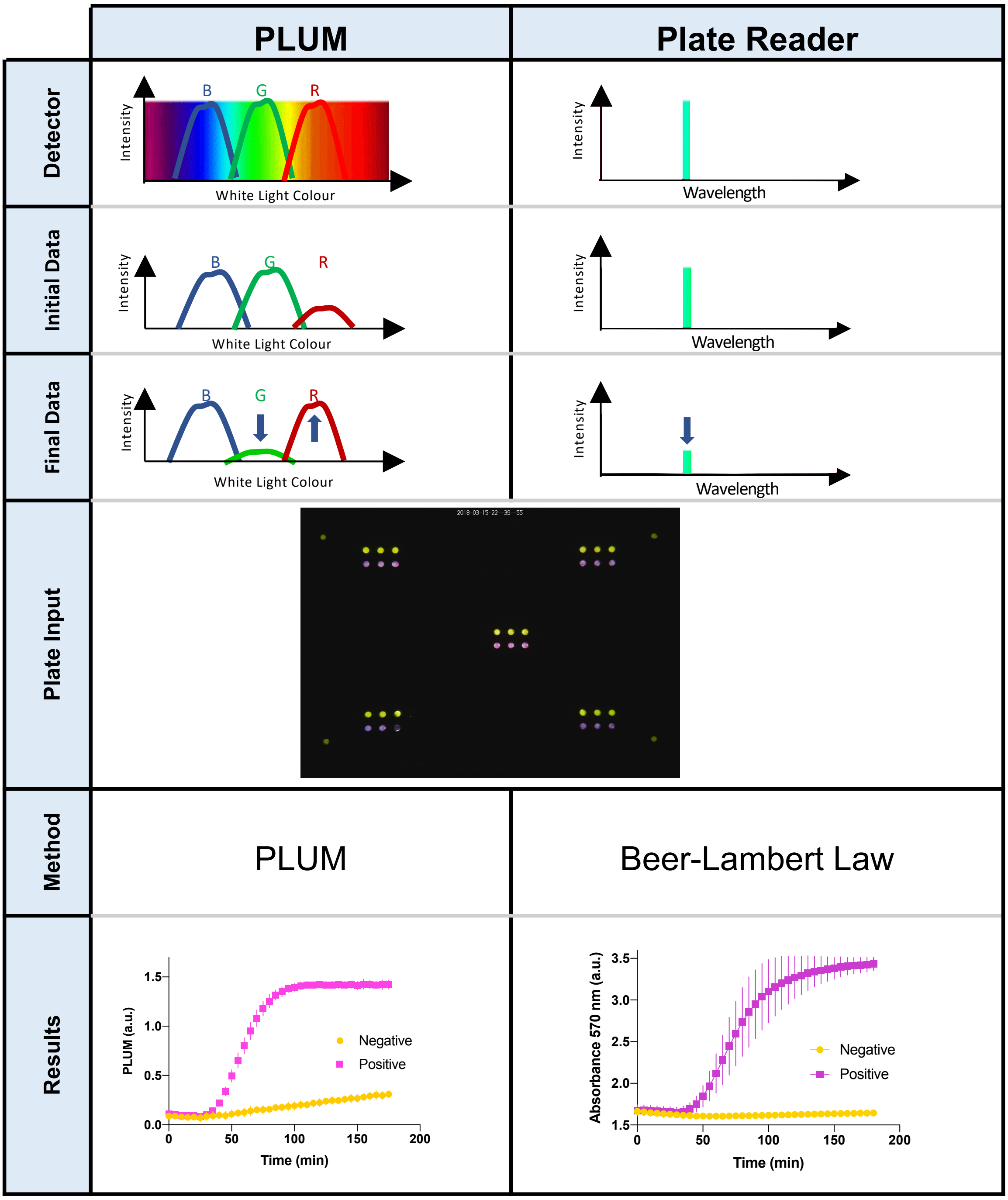

Supplementary Fig. 2. Comparison of quantitative colorimetric analysis methods for the PLUM reader (left panel) versus a conventional plate reader (right panel). PLUM (left column): Camera-based monitoring of changes in RGB channels for each of the 384 ROIs provides a summary of color changes in the form of an RGB ratio. The PLUM signal calculator software method places the increasing channel value over the decreasing channel value to provide absorbance-equivalent measurements that can be used to track changes in diagnostic reactions (blue/green). Plate reader (right column): Conventional plate readers monitor transmittance of a narrow band of wavelengths through diagnostic reactions (570 nm). As positive diagnostic samples turn purple, absorbance of the monitored wavelength leads to a decrease in transmitted light from the sample, which can be used to provide a quantitative output.

In order to assess the positional effect of reactions in PLUM, an experiment was performed in biological triplicate, each including a technical triplicate, at five different locations in a plate: four reactions close to the

edges of the plate and one at the center of the plate (plate input, and main Fig. 2f,g). Each reaction has a positive (purple) and a negative (yellow) sample. PLUM camera-based monitoring of reactions was performed in comparison to a conventional plate reader. As observed in the results (Fig. 2g, Supplementary Fig. 2), sample position in the plate did not introduce significant deviations to positive or negative measurements in either of the methods as demonstrated by the size of the SD. The graph (Fig. 2g) represents one of the biological experiments, including all its technical triplicates at the five different locations (so 15 positives and 15 negatives reactions are plotted). In this Supplementary Fig. 2, only one of the triplicate positive and negative measurements were plotted as the goal was to compare PLUM to a conventional reader.

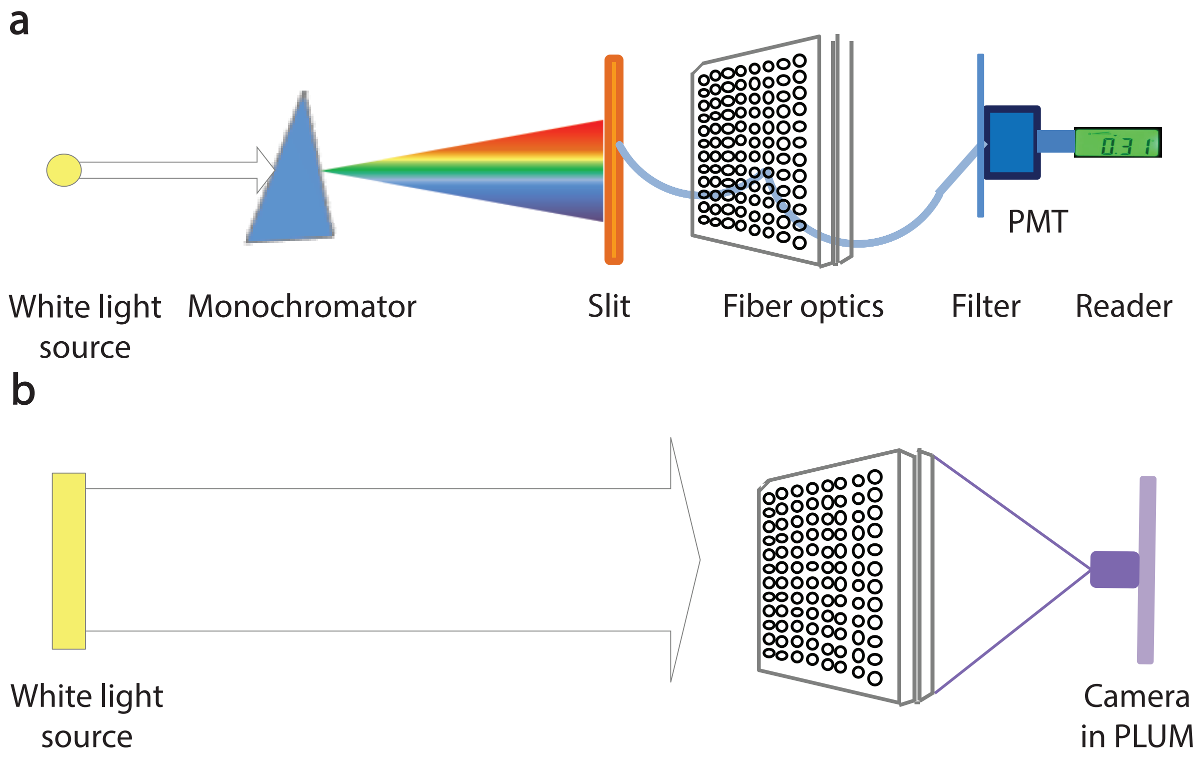

Supplementary Fig. 3. Schematic comparing the optical design of PLUM versus a conventional plate reader. **a**, A conventional plate reader is based on a movable spectrophotometer that is comprised of a monochromator, slit, and fiber optics. This optical monitoring apparatus moves across the plate at each read interval to illuminate and monitor light transmittance from wells. The collected light is filtered and quantified by a photomultiplier tube (PMT) to provide absorbance measurements via a digital reader. **b**, The PLUM device is composed of a white light source with a broad range of wavelengths that cover the visible spectrum and a camera to monitor changes in the light collected from samples. The light box provides even illuminance over the whole plate, which is placed at the focal point of the camera for collection of red, green and blue channel values of all 384 ROIs in parallel.

a

Samples	Ct value	Ct value
H_2O	N.A.	N.A.
DENV-1	N.A.	N.A.
DENV-2	N.A.	N.A.
DENV-3	N.A.	N.A.
DENV-4	N.A.	N.A.
YFV	N.A.	N.A.
ZIKV Am	12.40	12.52
ZIKV Af	N.A.	N.A.
CHIKV	N.A.	N.A.

b

Samples (PFU/mL)	Ct value	Ct value	Ct mean
H_2O	N.A.	N.A.	N.A.
10^{-3}	N.A.	N.A.	N.A.
10^{-2}	N.A.	N.A.	N.A.
10^{-1}	N.A.	N.A.	N.A.
10^{0}	N.A.	N.A.	N.A.
10^{1}	36.07	35.52	35.8
10^{2}	32.71	32.39	32.6
10^{3}	28.45	28.4	28.4
10^{4}	21.37	21.42	21.4
10^{5}	16.75	16.9	16.8

c

Time (min)	Threshold in PLUM a.u.	Accuracy	True Positive Samples	False Negative Samples	False Positive Samples	True Negative Samples	Total Samples
70	0.1316676	91.04%	66	7	17	178	268
75	0.1183366	91.04%	68	5	19	176	268
80	0.1280939	92.16%	68	5	16	179	268
85	0.1277769	89.18%	68	5	24	171	268
90	0.1386287	91.42%	68	5	18	177	268
95	0.141508	91.42%	68	5	18	177	268
100	0.1429698	95.15%	68	5	8	187	268
105	0.1616789	95.15%	70	3	10	185	268
110	0.144444	95.15%	68	5	8	187	268
115	0.1486262	96.27%	68	5	5	190	268
120	0.1631186	94.40%	68	5	10	185	268
125	0.1668622	97.39%	68	5	2	193	268
130	0.160333	98.51%	69	4	0	195	268

Supplementary Fig. 4. RT-qPCR data for Zika virus detection and threshold design. a, RT-qPCR data for analytical specificity of Zika virus detection. **b**, RT-qPCR data for analytical sensitivity of Zika virus detection. Zika virus concentration is reported as PFU/mL. For both (a) and (b), experiments were performed in biological triplicate, each containing a technical duplicate. Ct values displayed are technical duplicates of a representative experiment and Ct mean is the average of the technical duplicate. **c**, Threshold values of arbitrary unit (a.u.) for Zika virus diagnostics established using normalized readings from analytical sensitivity tests performed in PLUM device. Columns 1 and 2 present the time-based thresholds value determined in PLUM for Zika virus detection on sensitivity samples. Column 3 presents the overall accuracy of the detection in PLUM for patient sample data, compared to the RT-qPCR that was run in parallel, with performance metrics over time listed within columns 4 to 8.

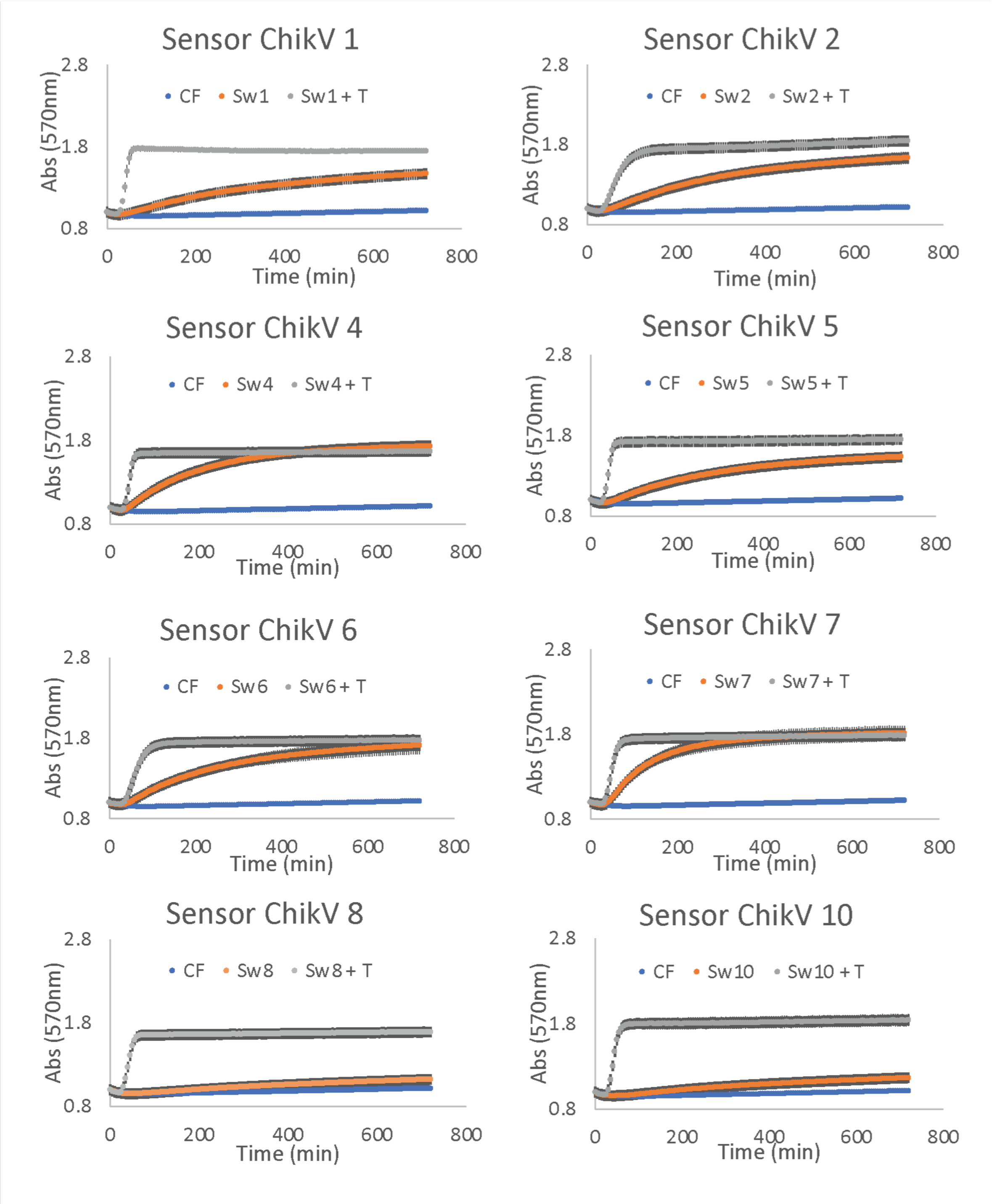

Supplementary Fig. 5. Data from screening of top performing candidate chikungunya virus toehold switch-based sensors. Time-course monitoring of LacZ expression (570 nm) from eight candidate toehold switch-based sensors (Sw; 33 nM) +/- synthetic trigger RNA (T) containing a fragment corresponding to the chikungunya viral genome (2 μM). Sensor ChikV 10 was the best performer and was the one used in the study. Each experiment was performed in biological triplicate, each containing a technical triplicate. Graphs are representative experiments of one of the biological triplicates using the mean of the technical triplicate +/- SD. Absorbance at 570 nm are normalized a t=0 and displayed as arbitrary units.

a

Samples	Ct value	Ct value
H2O	N.A.	N.A.
DENV-1	N.A.	N.A.
DENV-2	N.A.	N.A.
DENV-3	N.A.	N.A.
DENV-4	N.A.	N.A.
YFV	N.A.	N.A.
ZIKV Am	N.A.	N.A.
ZIKV Af	N.A.	N.A.
MAYV	N.A.	N.A.
CHIKV-PE	11.8	11.8
CHIKV-PB	10.8	11

b

Samples (PFU/mL)	Ct value	Ct value	Ct mean
H2O	N.A.	N.A.	N.A.
10^{-3}	N.A.	N.A.	N.A.
10^{-2}	N.A.	N.A.	N.A.
10^{-1}	36.4	37.4	36.9
10^{0}	33.6	33.5	33.6
10^{1}	30.6	30.5	30.6
10^{2}	27.3	26.9	27.1
10^{3}	23.2	23.1	23.2
10^{4}	19.5	19.8	19.7
10^{5}	15.6	15.5	15.6

c

Time (min)	Threshold in PLUM a.u.	Accuracy	True Positive Samples	False Negative Samples	False Positive Samples	True Negative Samples	Total Samples
75	0.08	98.46%	12	1	0	52	65
80	0.09	98.46%	12	1	0	52	65
85	0.07	98.46%	12	1	0	52	65
90	0.11	98.46%	12	1	0	52	65
95	0.11	98.46%	12	1	0	52	65
100	0.13	98.46%	12	1	0	52	65
105	0.14	98.46%	12	1	0	52	65
110	0.15	98.46%	12	1	0	52	65
115	0.15	98.46%	12	1	0	52	65
120	0.16	98.46%	12	1	0	52	65

Supplementary Fig. 6. RT-qPCR data for CHIKV detection and threshold design. a, RT-qPCR data for analytical specificity of chikungunya virus detection. **b**, RT-qPCR data for analytical sensitivity of chikungunya virus detection. Chikungunya virus concentration is reported as PFU/mL. For both (a) and (b), experiments were performed in biological triplicate, each containing a technical duplicate. Ct values displayed are technical duplicates of a representative experiment and Ct mean is the average of the technical duplicate. **c**, Threshold values of arbitrary unit (a.u.) for chikungunya diagnostics established using normalized readings in analytical sensitivity tests performed in PLUM device. Columns 1 and 2 present the time-based threshold values determined in PLUM for CHIKV detection on sensitivity samples. Column 3 presents the overall accuracy of the detection in PLUM for the patient samples data, compared to the RT-qPCR that was ran in parallel, with performance metrics over time listed within columns 4 to 8.

Table 1. Sequences.

**NASBA primers**	**Sequence**	**Length**
NASBA ZIKV_F	AATTCTAATACGACTCACTATAGGGAGAAGGGCACAGTGGGATGATCGTTA	51
NASBA ZIKV_R	CCTGTCCTCGGTTCACAATCAA	22
NASBA CHIKV_F	AATTCTAATACGACTCACTATAGGGAGAAGGCACACAACTGGTACTGCAGA	51
NASBA CHIKV_R	AATGGTGCTGTGTGCTGCAGCG	22
**Sensors**		
ZIKV sensor	TTTCGCTCTATTCTCATCAGTTTCATGTCCTGTGTCGGACTTTAGAACAGAGGAGATAAAGATGGACACAGGACACAACCTGGCGGCAGCGCAAAAG	97
CHIKV_s_TA_10	GCGCTAATACGACTCACTATAGGGCGTTCTTTTAGCCAATACTTGAAGCCAGATGGTGCCGTTATAGTTATGAACAGAGGAGACATAACATGAACGGCACCAACGCCGTTAACCTGGCGGCAGCGCAA	128
**Triggers**		
ZIKV trigger	CTAATACGACTCACTATAGGGCCAGCACAGTGGGATGATCGTTAATGACACAGGACATGAAACTGATGAGAATAGAGCGAAAGTTGAGATAACGCCCAATTCACCAAGAGCCGAAGCCACCCTGGGGGGGTTTGGAAGCCTAGGACTTGATTGTGAACCGAGGACAGGtagcataaccccttggggcctctaaacgggtcttgaggggttttttg	215
CHIKV trigger	GCGCTAATACGACTCACTATAGGGTTCATAGTGGGGCCAATGTCTTCAGCCTGGACACCTTTCGACAACAAAATCGTGGTGTACAAAGGCGACGTCTACAACATGGACTACCCGCCCTTCGGCGCAGGAAGACCAGGACAATTTGGCGACATCCAAAGTCGCACGCCTGAGAGCGAAGACATCTATGCTAACACACAACTGGTACTGCAGAGACCGTCCGCGGGTACGGTGCACGTGCCGTACTCTCAGGCACCATCTGGCTTCAAGTATTGGCTAAAAGAACGAGGGGCGTCGCTGCAGCACACAGCACCATTTGGCTGTCAAATAGCAACAAACCCGGTAAGAGCGATGAACTGC	357
T3T8 lentivirus trigger	GCCAGCACAGTGGGATGATCGTTAATGACACAGGACATGAAACTGATGAGAATAGAGCGAAAGTTGAGATAACGCCCAATTCACCAAGAGCCGAAGCCACCCTGGGGGGGTTTGGAAGCCTAGGACTTGATTGTGAACCGAGGACAGGactcgataaGCTGACCCTAATAGTGGCCATCATTTTGCTCGTGGCGCACTACATGTACTTGATCCCAGGGCTGCAGGCAGCAGCTGCGCGTGCTGCCCAGAAGAGAACGGCAGCTGGCATCATGAAGAACCCTGTTGTGGATGG	292
**PCR primers**		
in pCOLA-Duet backbone		
PCR sensor F	aattgactctcttccgggcg	20
PCR sensor R	AGTGTGACCGTGTGCTTCTC	20
in pET-15 backbone		
PCR trigger_F	CTCGACGCTCTCCCTTATGC	20
PCR trigger_R	Gcagtcaggcaccgtgtatgaaatc	25
dsDNA CHIKV trigger		
PCR CHIKV_F	GCGCTAATACGACTCACTATAGGGTTCATAGTGG	34
PCR CHIKV_R	GCAGTTCATCGCTCTTACCG	20

**qPCR primers**		
RT-qPCR ZIKV_F 1086	CCGCTGCCCAACACAAG	17
RT-qPCR ZIKV_R 1162c	CCACTAACGTTCTTTTGCAGACAT	24
RT-qPCR ZIKV_P 1107	FAM-AGCCTACCTTGACAAGCAGTCAGACACTCAA-MGB	39
RT-qPCR CHIKV_F	GCGCTAATACGACTCACTATAGGGTTCATAGTGG	34
RT-qPCR CHIKV_R	GCAGTTCATCGCTCTTACCG	20
RT-qPCR CHIKV_P 6919	FAM-AGGTACGCGCTTCAAGTTCGGCG	27
**Best performing CHIKV sensors**		
CHIKV_s_TA_01	GCGCTAATACGACTCACTATAGGGTTTTAGCCAATACTTGAAGCCAGATGGTGCCTGAGAGTTATAGTTATGAACAGAGGAGACATAACATGAACTCTCAGAACAGAGTTAACCTGGCGGCAGCGCAA	128
CHIKV_s_TA_02	GCGCTAATACGACTCACTATAGGGAAATTGTCCTGGTCTTCCTGCGCCGAAGGGCGGGTAGTTATAGTTATGAACAGAGGAGACATAACATGAACTACCCGAACGTAGTTAACCTGGCGGCAGCGCAA	128
CHIKV_s_TA_04	GCGCTAATACGACTCACTATAGGGAATACTTGAAGCCAGATGGTGCCTGAGAGTACGGCAGTTATAGTTATGAACAGAGGAGACATAACATGAACTGCCGTAACGCAGTTAACCTGGCGGCAGCGCAA	128
CHIKV_s_TA_05	GCGCTAATACGACTCACTATAGGGATAGATGTCTTCGCTCTCAGGCGTGCGACTTTGGATGTTATAGTTATGAACAGAGGAGACATAACATGAACATCCAAAACGATGTTAACCTGGCGGCAGCGCAA	128
CHIKV_s_TA_06	GCGCTAATACGACTCACTATAGGGACTTGAAGCCAGATGGTGCCTGAGAGTACGGCACGTGTTATAGTTATGAACAGAGGAGACATAACATGAACACGTGCAACCGTGTTAACCTGGCGGCAGCGCAA	128
CHIKV_s_TA_07	GCGCTAATACGACTCACTATAGGGAGCATAGATGTCTTCGCTCTCAGGCGTGCGACTTTGGTTATAGTTATGAACAGAGGAGACATAACATGAACCAAAGTAACTTGGTTAACCTGGCGGCAGCGCAA	128
CHIKV_s_TA_08	GCGCTAATACGACTCACTATAGGGCGGACGGTCTCTGCAGTACCAGTTGTGTGTTAGCATGTTATAGTTATGAACAGAGGAGACATAACATGAACATGCTAAACCATGTTAACCTGGCGGCAGCGCAA	128
CHIKV_s_TA_10	GCGCTAATACGACTCACTATAGGGCGTTCTTTTAGCCAATACTTGAAGCCAGATGGTGCCGTTATAGTTATGAACAGAGGAGACATAACATGAACGGCACCAACGCCGTTAACCTGGCGGCAGCGCAA	128

Table 2. Cost analysis of the diagnostic test.

	Product cost USD	Unit amount per product	Amount per reaction	Number of reactions per product	Cost / reaction - USD
NASBA	254.00	480 uL	5.0000 uL	96.00	2.65
NASBA primers	8.52	800 uL	0.1000 uL	8000.00	0.00
RNAse inhibitors NASBA	51.85	75 uL	0.0500 uL	1500.00	0.03
384 well plate (1 well)	1157 / pack	19,200 wells	1 well	19200.00	0.06
PURExpress	2377.04	2500 uL	2.67 uL	936	2.54
Linear DNA sensor 25 ug for 3300 bp	687.7	25 uL	0.0069 uL	3637	0.19
RNAse inhibitors CF	51.85	75 uL	0.0133 uL	5639.10	0.01
CPRG (25 ug/uL)	85.93	10,000 uL	0.1920 uL	52083.33	0.00
Paper Disc	0.20			300.00	0.00
**TOTAL cost per reaction**					**5.48**

Note 1: Toehold switch background information

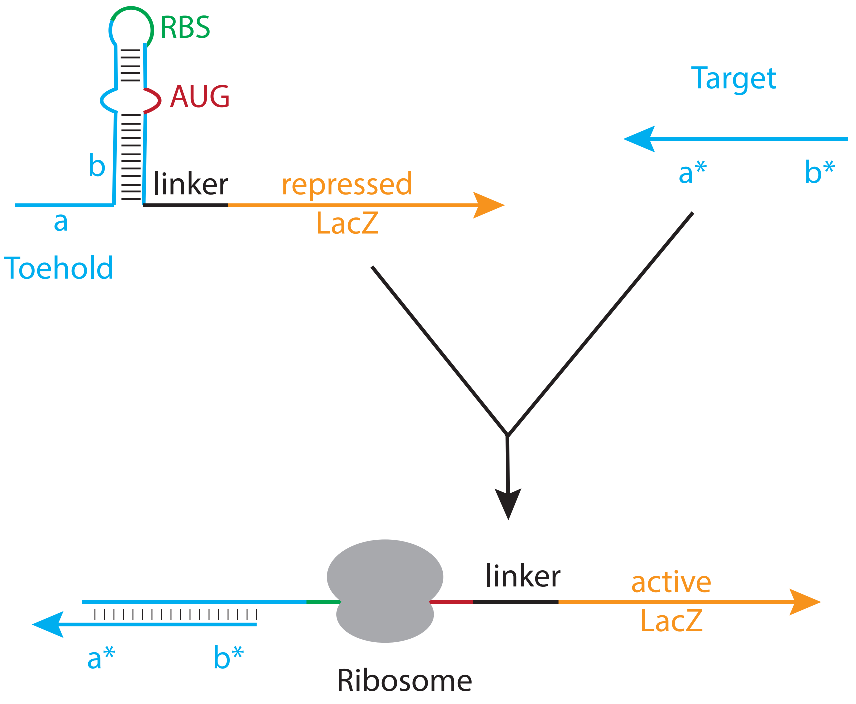

Toehold switches are a novel class of riboregulators described for the first time in 2014[1]. These programmable regulators enable post-transcriptional activation of protein translation through linear-linear RNA interactions and subsequent strand displacement.

Toehold switches contain:

- Defined regions
  - Ribosomal binding site (RBS) of the mRNA, sequestered in the hairpin
  - AUG start codon, sequestered in the hairpin
  - 21-nt linker between the hairpin and the regulated gene
  - The sequence of the gene being regulated (e.g., LacZ)
- Target-dependent region:
  - A sequence specific region (a,b), complementary to a corresponding trigger RNA (a*,b*)

The riboregulator folds into the hairpin secondary structure depicted in the schematic above and acts as a repressor of translation. In this conformation, the RBS as well as the start codon are sequestered into the hairpin and are not accessible to the ribosome.

Upon binding to its sequence specific trigger RNA (a*,b*), the toehold switch linearizes, releasing the RBS sequence and enabling expression of the downstream gene, here LacZ.

Moreover, the toehold switch mechanism is able to tolerate up to 4-nt mismatches between the target sequence and 25-nt toehold domain, allowing it to accommodate the intrinsic virus sequence variability (~11%)[2].

Method 1: RNA copy number calculation

In order to determine the RNA copy number per μL used in each NASBA reaction, the following example calculation was used:

Example calculation of the copy number of synthetic RNA molecule of Zika virus.
$[C] = A_{260} / \varepsilon$
Where [C] represents the concentration of the solution, A_{260} represents the absorbance at 260 nm of the solution, and where ε represents the extinction coefficients of the molecule present in the solution (here, the synthetic RNA).
ε values for the Zika virus synthetic RNA or chikungunya virus synthetic RNA
ε_{ZIKV} = 1,506,900 L / (mol x cm)
ε_{CHIKV} = 3,433,100 L / (mol x cm)

Following *in vitro* RNA synthesis, absorbance of the solution at 260 nm was determined using a nanodrop.
In this case, A_{260} = 3.10
So [C] = 3.10 / 1,506,900 = 2.057 μM
[C] can also be expressed as:
[C] = n / V
Where n is the moles (mol) and V is the volume of the system expressed in liters (L).
So n = [C] x V.
And in 1 μL, n = 2.057 x 10^{-6} x 10^{-6} = 2.057 x 10^{-12} mol.
Finally, the number of molecules (m) per mol:
m = n x A
Where A is the Avogadro number and A = 6.022 x 10^{23} molecules / mol.
In 1 μL, m = 2.057 x 10^{-12} x 6.022 x 10^{23} = 1.24 x 10^{12} molecules.
Serial dilutions of the solution where performed, from 1.24 x 10^{6} molecules / μL to 1.24 molecules / μL (or 1.239 x 10^{0} molecules / μL).

Method 2: Documentation for international field work

The following information and documents are included as an example of what our team prepared in planning for international field work. This information is provided as a guide, and requirements for importation/exportation of instruments, reagents, and samples may vary from country to country. Further, in some cases, the documents we prepared were required and in others, they were not necessary. Documents S1.2-1.6 were kept on person during travel and duplicate copies were stored in the luggage. We include templates for these documents that can be used as a guide when preparing for international field work. It is important to always check the local laws and requirements for customs before travelling.

In this section, the “Guest” refers to the research team (and/or their institution) travelling to conduct the field work and “Host” refers to the researchers (and/or their institution) in the country where the field work was conducted.

Table S1: List of documents kept on person for travel with scientific instruments and reagents

File ID	**Document**	**Justification**	**Prepared by**	
			Guest	Host
S1.1	Ethics approval	Work involving collection of patient samples requires research ethics board approval.	x	x
S1.2	Letter of donation	Explains that the consumables and reagents used in the field work constitute a donation from the Guest institution to the Host institution, that the nature of the work is non-commercial, and that no money was given in consideration.	x	
S1.3	Letter of importation	Explains what the equipment, consumables, and reagents are and the purpose of the field work. One copy in English and one copy in the Host country’s official language.		x
S1.4	Bill of materials (BOM) for PLUM	Details the components of the PLUM instrument and gives the costs of each item.	x	
S1.5	Manifest of equipment, consumables, and reagents	An itemized inventory to account for all the laboratory materials travelling to the Host country. The items listed should correspond with the Letter of donation.	x	
S1.6	Supporting documents	Past research papers, press releases, grant award letters, and other documents to demonstrate that the field work is part of sustained and legitimate academic research work.	x	

S1.2 Letter of Donation Template

(On Guest institutional letterhead)

From: Associate Dean of Research (or equivalent role) at Guest institution

Certificate of Donation

Please be advised that the equipment and supplies listed on the attached document constitute a gift to [Host institution] in [city, country] from [Guest institution] in [city, country] for the ongoing research project entitled "[title of research project]."

It is my understanding that the items will be used for the express purpose of [...] by [Host institution] and their collaborating partners. The items will be brought in by [name(s) of traveller(s)], a [role of traveller(s)] as carry-on and check-in luggage [date of flight]. As outlined in the attached document, the consumables will be used during experiments by the team at [Host institution] and the [names of instruments] will return to [Guest country] at the end of the trip.

No money was received or given for these items. These items will not be resold by [Host institution] and have no commercial value.

If you have any questions concerning the donation, please feel free to e-mail me at [email address].

Sincerely,

[Signature]

S1.3 Letter of importation

(On Host institutional letterhead)

(Duplicate copy in Host country official language)

From: Host Principal Investigator, Department Chair, or Faculty Dean

To Whom It May Concern:

[Introduction to who the writer is, their role in the research project, the title of the research project, and the dates of the research project. Identification of the travellers from the Guest institution and their role in the research project. Identification of the funding and sponsoring organizations.]

[Lay background of the research project and its relationship to the Host institution's work. Description of the equipment, reagents, and consumables being brought into the country.]

[Request for permission to bring the items into the country. Declaration of the safety and non-toxicity of the reagents. Declaration of the non-commercial value and research-only use of the transported equipment and reagents.]

Sincerely,

[Signature]

S1.4 Bill of Materials (BOM) for PLUM instrument

PART	VENDOR SPECIFICATION/CODE	VENDOR	COST (CAD)
Incubator	INCUKIT™ MINI	Incubator Warehouse	$ 62.49
Strip LEDs	YJ-VTC-RB-5730-12V-56	YUJI LED	$ 48.88
Servo Motor (Continous Rotation)	HSR-1425CR	Robotshop	$ 21.51
Servo Gear	RB-Sct-458	Robotshop	$ 20.18
Temperature Sensor	DS18B20	Robotshop	$ 8.85
DC-DC Converter to 5V	5V/3A Step Down Voltage Module 12V to 5V Power Supply with USB Interface	Amazon	$ 12.99
Micro USB to Mini USB 2.0 Converter	StarTech.com Micro USB to Mini USB 2.0 Adapter M/F UUSBMUSBMF	Amazon	$ 6.99
DC-DC Converter 5-11V to 12V 2A	DIGITEN DC 6V 5-11V to 12V 2A Step up Converter Regulator	Amazon	$ 14.19
Keyboard		Amazon	$ 19.99
Mouse	Edota 2.4G Professional Rechargeable Wireless Gaming Mouse	Amazon	$ 11.99
Power Bar	12 Volt Power Supply - 7 Amp Standard (12V 7A DC) Adapter	Amazon	$ 25.51
Power Adapter Head		Digikey	$ 1.40
Black Acrylic for enclosure	Black 3mm thick Acrylic by 4 foot by 8 foot cut in 12 by 20	McMaster Carr	$ 21.41
Clear Acrylic	8560K171	McMaster Carr	$ 1.32
Diffuser Sheets (Matte dura-lar and matte mylar)		Above ground art supplies	$ 3.29
Raspi 3	Raspberry Pi 3 Model B Board	Amazon	$ 52.99
Raspi Camera	Raspberry Pi Camera Module V2 - 8 Megapixel,1080p	Amazon	$ 32.99
Flex Cable	Flex Cable for Raspberry Pi Camera - 24" / 610mm	Amazon	$ 7.99
Raspi Touch Screen	TOUCHSCREEN LCD DISPLAY	Amazon	$ 102.95
Solderless Connectors	LED Light Strip Connector	Amazon	$ 5.00
SD Card	Extreme Pro 32GB	Amazon	$ 45.03
Screws for display		McMaster Carr	$ 3.86
Nuts for display		McMaster Carr	$ 1.00
Hex Stand-off 18-8 Stainless Steel, 1/4" Hex, 1-3/4" Long, 4-40 Thread Size		McMaster Carr	$ 10.68
Hex stand-off 6/6 Nylon, 1/4" Hex Size, 5/8" Long, 4-40 Thread Size		McMaster Carr	$ 12.24
4-40 screw 3/16"		McMaster Carr	$ 0.83
4-40 screw 3/8"		McMaster Carr	$ 0.90
4-40 screw 7/16"		McMaster Carr	$ 0.15
4-40 screw 1/2"		McMaster Carr	$ 1.01
18-8 Stainless Steel Hex Nut		McMaster Carr	$ 0.29
Rocker Switch		McMaster Carr	$ 1.26
Power Plug		McMaster Carr	$ 1.07
Angle brackets		Digikey	$ 6.40
3d print parts		Shop3d	$ 2.40
PCB		Active Surplus	$ 1.95
Adafruit t-cobbler head		Adafruit	$ 9.94
Wires			$ 12.00
Microswitch		Digikey	$ 6.50
Connectors			$ 4.50
Solder			$ 1.00

S1.5 Manifest of equipment, consumables, and reagents

Materials, Reagents, Equipment List				
**Item**				
**Materials**	**Quantity Recife**	**Quantity Toronto**	**Product ID**	**Vendor**
Corning 384 Well Plates		1 case (50)	CLS3544	Sigma Aldrich
Aluminum Sealing Tape		1 box	95.1995	Sarstedt
PCR Tape		1 box	95.1994	Sarstedt
Biopsy punches		1 box of 15-20	33-31-P/25	McKesson
Tweezers		4	Non-surgical,ex	Amazon
Cutting Pad		3	Any, ex:	Amazon
blades		3		
Whatman® quantitative filter paper, ashless, Grade 42		2 boxes	WHA1442042	Sigma Aldrich
yellow acrylic markers		a tube		
PCR Tubes (Strip/Individual) (0.2mL)			Any	
1.5mL Tubes	1500		Any	
50mL Falcon Tubes	20 tubes		Any	
QIAamp Viral RNA Mini Kit			#52904/52906	Qiagen
Qiagen PCR purification kits	2		28106	Qiagen
Q5 Polymerase		2	M0491L	New England Biolabs
dNTP set		2	N0446S	New England Biolabs
T7 transcribe-RNA Synthesis kit		2	E2040S	New England Biolabs
**Reagents**				
BSA	0	w filter paper	CAS#9048-46-8	Bioshop Canada
CPRG	1	1	10884308001	Sigma Aldrich
NASBA Liquid Kits		14 kits	SKU: NWK-1	Life Sciences Advanced Technologies Inc.
Rnase Inhibitor		3	M0314S	New England Biolabs
NEB PURExpress kits		5 kits + 2	-	
Nuclease Free Water			Any	
**Equipment**				
Thermal Cyclers	2			
Nanodrop/Nucleic Acid quantification equipment				
PLUM	1 to 2	1	-	
**DNA**				
NASBA Primers	2	3		
qPCR Primers		3		
Trigger Linearization Primers		3		
Switch Linearization Primers	2	3		
Switch Linear DNA	50ug	Linearization for 50-75 (2-3 DAYS) PCR		
Switch Plasmid DNA	10ug	1 miniprep		
Trigger Plasmid DNA	10ug	1 miniprep		

S1.6 Supporting documents

To provide support for the legitimate nature of our research project, we brought along copies of the following documents:

- Previous publications relating to the ongoing research
- Funding announcements
- Press releases and news stories related to the project
- Printed slides from research presentations

Note 2: Hardware: Supplemental Files for Electronic Optical Reader

A. Original Manufacturing files can be accessed here as a zip file (https://github.com/PardeeLab/zikaproject_hardware.git) with the following information:
  a. Laser Cut Files.
  b. 3D Print Files.
  c. KiCad Circuit Board Files.
  d. PLUM Assembly FreeCAD File.
  e. PLUM Bill of Materials (BOM).

B. Motherboard Schematic and 3D View

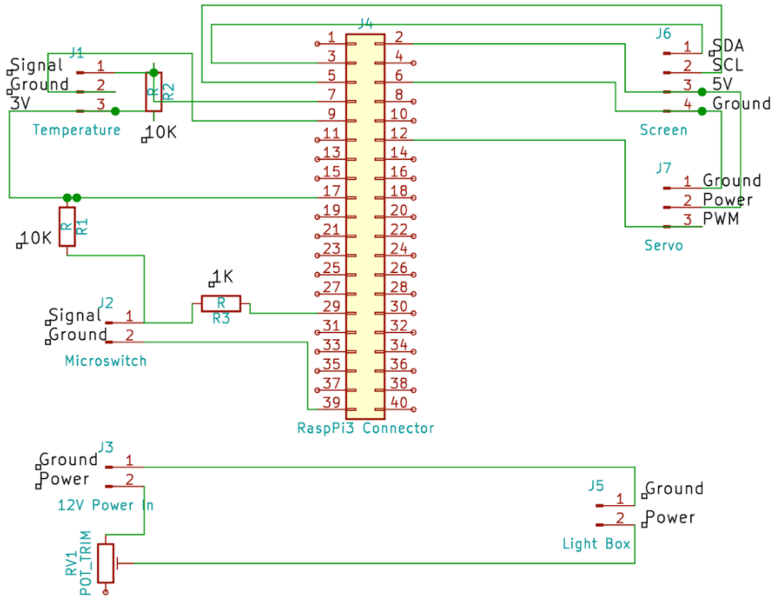

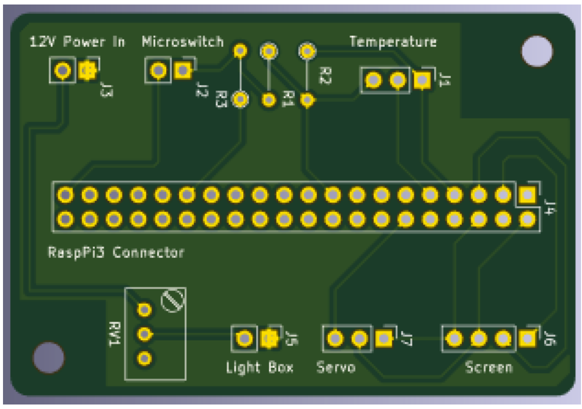

C. Backlit Lightbox Controller Schematic and 3D View

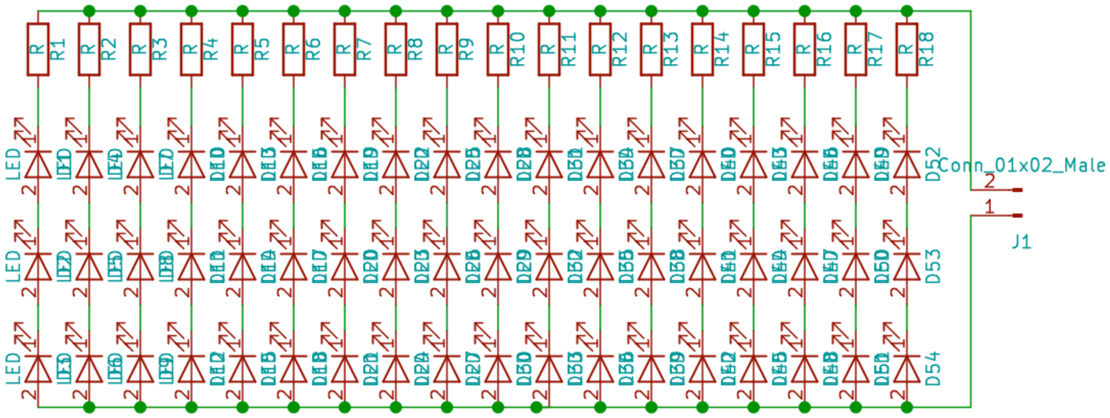

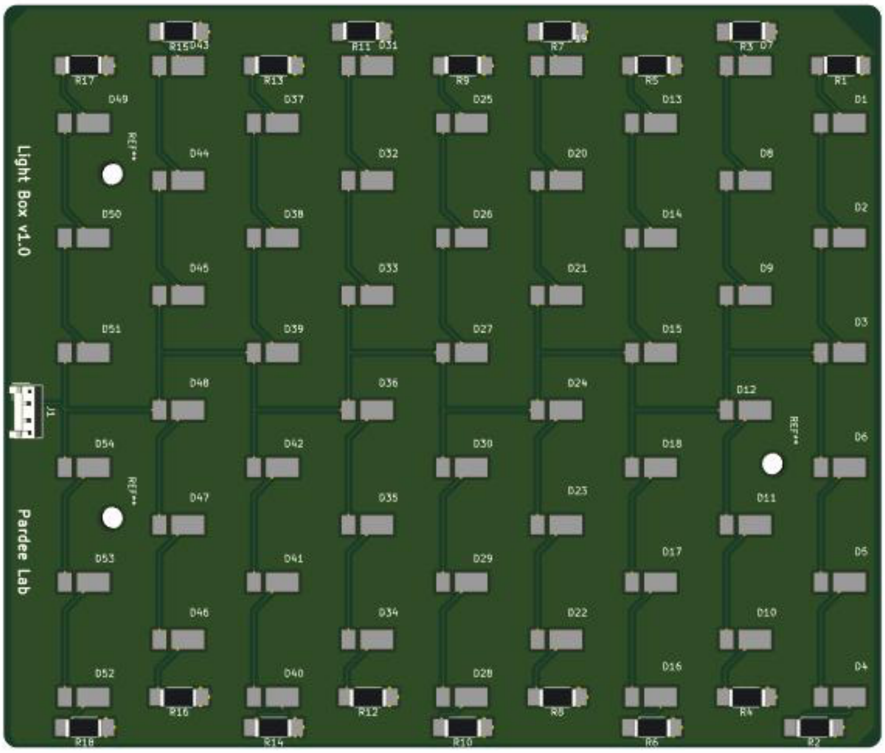

D. 3D Printed Part Cost

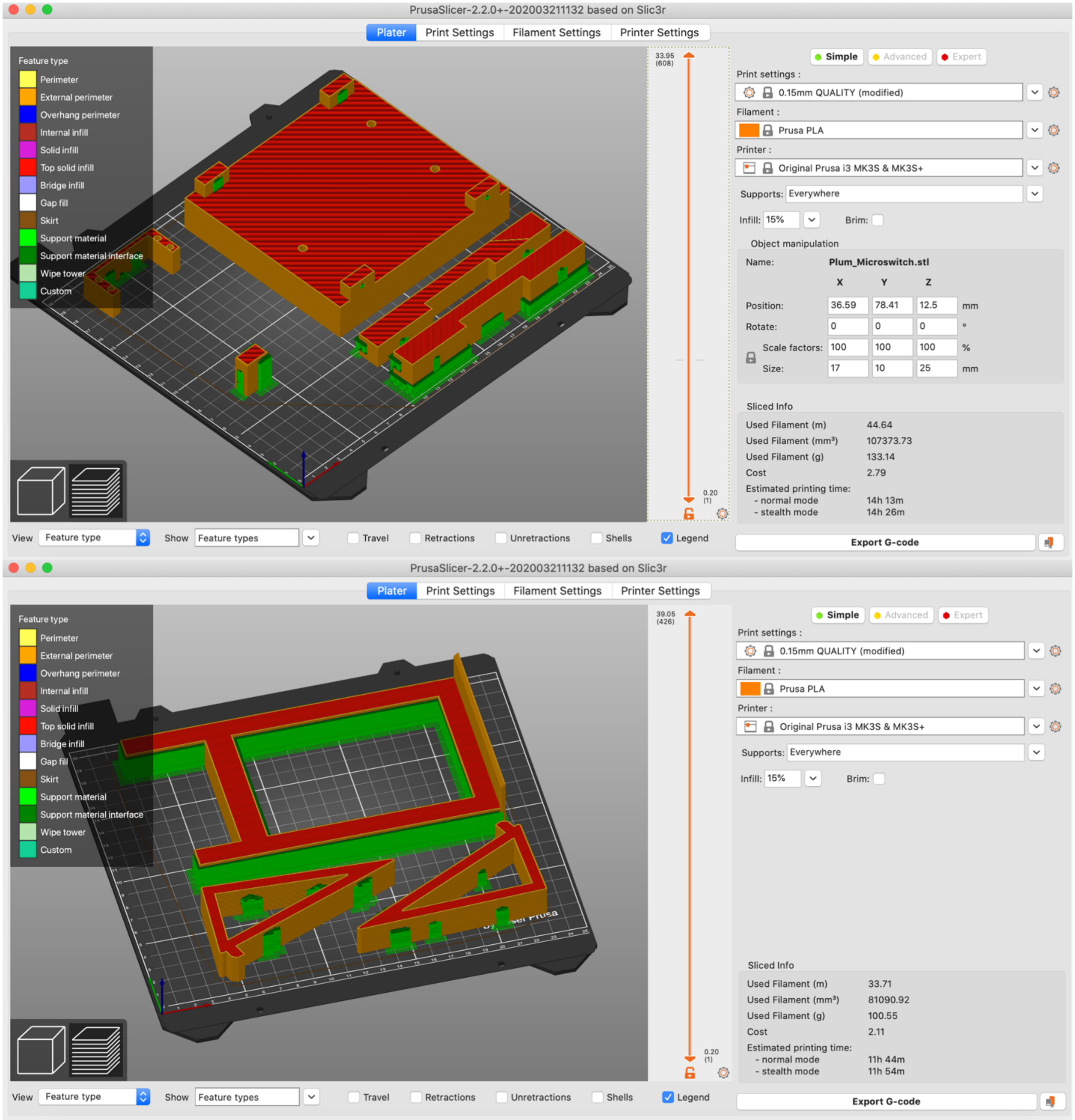

References

1. Green, A. A., Silver, P. A., Collins, J. J. & Yin, P. Toehold switches: De-novo-designed regulators of gene expression. *Cell* (2014) doi:10.1016/j.cell.2014.10.002.
2. Pardee, K. *et al.* Rapid, Low-Cost Detection of Zika Virus Using Programmable Biomolecular Components. *Cell* **165**, 1255–1266 (2016).
