# Supplementary material for: Field validation of the performance of paper-based tests for the detection of the Zika and chikungunya viruses in serum samples
Source: Nat Biomed Eng. 2022 Mar 7;6(3):246–56. doi: 10.1038/s41551-022-00850-0 (PMC8940623; doi:10.1038/s41551-022-00850-0)
Supplement: Supplementary file 2 — Reporting Summary [file 41551_2022_850_MOESM2_ESM.pdf]

nature portfolio

Corresponding author(s): Keith Pardee

Last updated by author(s): Dec 23, 2021

Reporting Summary

Nature Portfolio wishes to improve the reproducibility of the work that we publish. This form provides structure for consistency and transparency in reporting. For further information on Nature Portfolio policies, see our Editorial Policies and the Editorial Policy Checklist.

Statistics

For all statistical analyses, confirm that the following items are present in the figure legend, table legend, main text, or Methods section.

n/a	Confirmed	
☐	☒	The exact sample size (n) for each experimental group/condition, given as a discrete number and unit of measurement
☐	☒	A statement on whether measurements were taken from distinct samples or whether the same sample was measured repeatedly
☐	☒	The statistical test(s) used AND whether they are one- or two-sided<br>*Only common tests should be described solely by name; describe more complex techniques in the Methods section.*
☒	☐	A description of all covariates tested
☒	☐	A description of any assumptions or corrections, such as tests of normality and adjustment for multiple comparisons
☐	☒	A full description of the statistical parameters including central tendency (e.g. means) or other basic estimates (e.g. regression coefficient) AND variation (e.g. standard deviation) or associated estimates of uncertainty (e.g. confidence intervals)
☐	☒	For null hypothesis testing, the test statistic (e.g. F, t, r) with confidence intervals, effect sizes, degrees of freedom and P value noted<br>*Give P values as exact values whenever suitable.*
☒	☐	For Bayesian analysis, information on the choice of priors and Markov chain Monte Carlo settings
☒	☐	For hierarchical and complex designs, identification of the appropriate level for tests and full reporting of outcomes
☒	☐	Estimates of effect sizes (e.g. Cohen's d, Pearson's r), indicating how they were calculated

Our web collection on statistics for biologists contains articles on many of the points above.

Software and code

Policy information about availability of computer code

Data collection	The experimental data were collected using custom code, available on Github at https://github.com/PardeeLab/zikaproject_plumcode.git.
Data analysis	The logistic test was performed using STATA 15. The accuracy test was performed using https://www.medcalc.org/calc/diagnostic_test.php.<br>Data analyses on the PLUM reader were performed using custom code available at https://github.com/PardeeLab/zikaproject_plumcode.git.

For manuscripts utilizing custom algorithms or software that are central to the research but not yet described in published literature, software must be made available to editors and reviewers. We strongly encourage code deposition in a community repository (e.g. GitHub). See the Nature Portfolio guidelines for submitting code & software for further information.

Data

Policy information about availability of data

All manuscripts must include a data availability statement. This statement should provide the following information, where applicable:

- Accession codes, unique identifiers, or web links for publicly available datasets
- A description of any restrictions on data availability
- For clinical datasets or third party data, please ensure that the statement adheres to our policy

The main data supporting the results in this study are available within the paper and its Supplementary Information. All the experimental raw data are available for research purposes from the corresponding author on reasonable request.

Field-specific reporting

Please select the one below that is the best fit for your research. If you are not sure, read the appropriate sections before making your selection.

☒ Life sciences ☐ Behavioural & social sciences ☐ Ecological, evolutionary & environmental sciences

For a reference copy of the document with all sections, see nature.com/documents/nr-reporting-summary-flat.pdf

Life sciences study design

All studies must disclose on these points even when the disclosure is negative.

Sample size	Aliquots of serum were taken from sera already collected from the patient for medical diagnosis. From these subpopulations, we assumed a 50% prevalence, as measured by qPCR. Targeting 95% sensitivity and specificity for both assays and a lower bound of 80% for the 95% confidence interval, we calculated a minimum sample size of 70 for each assay. A total of 268 samples were collected for Zika, and 65 samples were collected for chikungunya.
Data exclusions	No data were excluded.
Replication	All in vitro studies were independently performed in triplicates. The field trial on serum of patient samples was only performed once, and it compared during the same day the diagnostic system to gold-standard RT-qPCR.
Randomization	Positive and negative serum samples were randomly placed on ice.
Blinding	The experimenters were blinded while performing the field trial.

Reporting for specific materials, systems and methods

We require information from authors about some types of materials, experimental systems and methods used in many studies. Here, indicate whether each material, system or method listed is relevant to your study. If you are not sure if a list item applies to your research, read the appropriate section before selecting a response.

Materials & experimental systems

n/a	Involved in the study	
☒	☐	Antibodies
☒	☐	Eukaryotic cell lines
☒	☐	Palaeontology and archaeology
☒	☐	Animals and other organisms
☐	☒	Human research participants
☒	☐	Clinical data
☒	☐	Dual use research of concern

Methods

n/a	Involved in the study	
☒	☐	ChIP-seq
☒	☐	Flow cytometry
☒	☐	MRI-based neuroimaging

Human research participants

Policy information about studies involving human research participants

Population characteristics	The participants independently chose to seek medical attention from enrolled clinics. Upon physician assessment, patients with a high clinical index of suspicion for either Zika or chikungunya viral infections were voluntarily enrolled in the study by a third party (non-medical staff) and with informed consent.
Recruitment	Patient samples were obtained from suspected arbovirus infections, who presented arthralgia, fever, exanthema and other related symptoms in an endemic area of several arboviruses in Latin America.
Ethics oversight	This study was approved by the Fiocruz-PE Institutional Review Board (IRB) under protocol 80247417.4.0000.5190 and REB approval number at the University of Toronto (Protocol# 39531), and was conducted in accordance with relevant regulations and guidelines, including the ethical principles for medical research involving human subjects designed by World Medical Association Declaration of Helsinki.

Note that full information on the approval of the study protocol must also be provided in the manuscript.
